# High‐Throughput Electromechanical Coupling Chip Systems for Real‐Time 3D Invasion/Migration Assay of Cells

**DOI:** 10.1002/advs.202300882

**Published:** 2023-04-23

**Authors:** Nan Jiang, Liang Xu, Yiming Han, Shuyi Wang, Xiaocen Duan, Jingyao Dai, Yunxing Hu, Xiaozhi Liu, Zhiqiang Liu, Jianyong Huang

**Affiliations:** ^1^ Department of Mechanics and Engineering Science, and Beijing Innovation Center for Engineering Science and Advanced Technology College of Engineering Peking University Beijing 100871 P. R. China; ^2^ Academy for Advanced Interdisciplinary Studies Peking University Beijing 100871 P. R. China; ^3^ Department of Hepatobiliary Surgery Air Force Medical Center Beijing P. R. China 100142; ^4^ Tianjin Key Laboratory of Epigenetics for Organ Development of Premature Infants Fifth Central Hospital of Tianjin Tianjin 300450 P. R. China; ^5^ Department of Physiology and Pathopgysiology School of Basic Medical Sciences Tianjin Medical University Tianjin 300070 P. R. China

**Keywords:** 3D cell migration and invasion, complex impedance spectra, drug screening, electromechanical coupling chip, mechanobiology

## Abstract

Cell invasion/migration through three‐dimensional (3D) tissues is not only essential for physiological/pathological processes, but a hallmark of cancer malignancy. However, how to quantify spatiotemporal dynamics of 3D cell migration/invasion is challenging. Here, this work reports a 3D cell invasion/migration assay (3D‐CIMA) based on electromechanical coupling chip systems, which can monitor spatiotemporal dynamics of 3D cell invasion/migration in a real‐time, label‐free, nondestructive, and high‐throughput way. In combination with 3D topological networks and complex impedance detection technology, this work shows that 3D‐CIMA can quantitively characterize collective invasion/migration dynamics of cancer cells in 3D extracellular matrix (ECM) with controllable biophysical/biomechanical properties. More importantly, this work further reveals that it has the capability to not only carry out quantitative evaluation of anti‐tumor drugs in 3D microenvironments that minimize the impact of cell culture dimensions, but also grade clinical cancer specimens. The proposed 3D‐CIMA offers a new quantitative methodology for investigating cell interactions with 3D extracellular microenvironments, which has potential applications in various fields like mechanobiology, drug screening, and even precision medicine.

## Graphical abstract

In vitro characterization of 3D cell migration/invasion still poses multiple challenges. With the aid of 3D topological networks and complex impedance detection strategies, this paper develops electromechanical coupling chip systems, which are not only competent for quantifying spatiotemporal dynamics of 3D cell migration/invasion and quantitative screening of anti‐tumor drugs, but also capable of grading clinical cancer specimens.

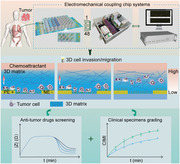

## 1 Introduction

Metastasis‐associated cell migration and invasion lead to the formation of secondary tumors and thus induce organ failure, which is responsible for ~90% of tumor‐caused deaths.^[^ ^1^, ^2^ ^]^ In essence, cell migration and invasion involve a multistep process composed of a variety of biochemical, biophysical and mechanobiological events related to cellular functions and behaviors.^[^ ^3^, ^4^ ^]^ It has been identified that there exist at least four typical migratory modes, that is, mesenchymal, amoeboid, lobopodial, and collective migration, which can flexibly interconvert between these modes relying on a cascade of signal transduction pathways regulated by intracellular components, like actin cytoskeletons and small GTPases, and extracellular microenvironments with chemical, physical, geometrical and mechanical cues.^[^ ^5^, ^6^, ^7^ ^]^ At present, most of in vitro experimental studies are focused on two‐dimensional (2D) migration and invasion behaviors of cells on flat substrates made from glass, plastic or polyacrylamide gels, which has already shed light on some key biomolecular and biophysical mechanisms of directional migration and invasion in response to extracellular stimuli such as chemical gradients of chemokines,^[^ ^8^ ^]^ substrate rigidities,^[^ ^9^ ^]^ and electrical fields.^[^ ^10^ ^]^ Nevertheless, cell migration and invasion are essentially 3D in the complex native environments of living tissues.^[^ ^5^ ^]^ Different from the 2D situation, the 3D migration and invasion through ECM or tissues normally require cells to navigate complex extracellular micro‐surroundings with barriers to locomotion, thereby causing the cell bodies and even nuclei to be mechanically squeezed.^[^ ^11^ ^]^ This process is usually accompanied by a sequence of biochemical, biophysical and mechanobiological signal transduction events, and thus prompt the cells to perceive externally spatial dimension‐related information, which can in turn modulate their functions and behaviors.^[^ ^12^, ^13^ ^]^ There is increasing evidence that, in comparison with 2D migration and invasion on flat surfaces, those in three dimensions are more easily regulated by ECM properties such as ECM compositions, pore sizes, level of crosslinking, stiffness and biodegradability,^[^ ^14^ ^]^ so that they have to optimize their migratory/invasive strategies through modifying cell adhesions with adjacent cells or ECM, actin protrusion/hydrostatic membrane blebs, actomyosin contraction, nuclear deformability, proteolytic capacity, etc.^[^ ^15^, ^16^, ^17^ ^]^ One typical example is that, unlike those in the 2D microenvironments, the cells undergoing 3D migration and invasion likely reduce the dependence upon adhesive interactions with surrounding ECM and simultaneously secrete active matrix metalloproteinases (MMPs), particularly membrane‐tethered (MT)1‐MMP/MMP‐14 as the key enzyme, which may break down ECM macromolecules and thus create macroscopic cavities to promote their movement.^[^ ^18^, ^19^, ^20^ ^]^

3D‐matrix‐based in vitro models offer an attractive and effective tool to dissect 3D migration and invasion of cells, which can bridge the gap between traditional 2D in vitro cell culture and animal models.^[^ ^2^, ^21^ ^]^ 3D in vitro models based on microfluidic devices, for instance, allow us to more closely mimic complicated tumor microenvironment (TME) with physiologically and pathologically relevant biological and biophysical cues, which can therefore be employed to explore cancer cell migration and invasion through TME under well‐controlled pathophysiological conditions modulated by tumor progression events.^[^ ^22^, ^23^ ^]^ Recent progresses indicate that, in combination with advanced optical imaging technology, microfluidic‐based 3D in vitro models including cancer‐on‐a‐chips and organ‐on‐a‐chips, are a powerful tool to quantitatively investigate the regulatory mechanisms of ECM/TME factors on 3D migration and invasion, for example, ECM rigidities, interstitial and shear flow, metabolic gradients, hypoxia and cytokine.^[^ ^24^, ^25^, ^26^, ^27^ ^]^

Generally, the 3D‐matrix‐based in vitro models are more similar to native ECM/TME than traditional 2D in vitro models utilized to assess cell migration and invasion.^[^ ^28^ ^]^ On the other hand, in comparison with expensive and time‐consuming animal models that can mimic various complex microenvironments in vivo, these 3D‐matrix‐based models are not only simple and easy to implement, but also facilitate the integration of various ECM/TME‐related factors in a quantitatively controllable manner. Nevertheless, how to characterize spatiotemporal dynamics of cell migration/invasion in 3D microenvironments in a real‐time, label‐free, nondestructive and high‐throughput fashion still poses multiple challenges in practice. The classical transwell‐based migration/invasion assay, which employs chemokine gradients between upper and lower chambers to drive cell invasion/migration, can only achieve an end‐point, semiquantitative, and label‐based characterization of cell migration and invasion,^[^ ^29^ ^]^ whereas the conventional interdigitated electrode array (IDEA) is usually difficult to quantitatively detect the 3D cell migration and invasion in a way of high spatiotemporal resolution.^[^ ^30^, ^31^, ^32^ ^]^ Here, we aim to develop a set of 3D cell invasion/migration assay (3D‐CIMA) on the basis of electromechanical coupling chip systems. It allows us to quantify spatiotemporal dynamic processes of 3D cell migration and invasion in a real‐time, label‐free, nondestructive, and high‐throughput manner. With the aid of 3D topological networks of collagen I (Coll I) with controllable biophysical/biomechanical properties and well‐established complex impedance detection strategies, the proposed 3D‐CIMA can be adopted to quantitatively detect migratory and invasive phenotypes regulated by the 3D ECM/TME and mechanobiological responses to pathophysiologically relevant biochemical and biophysical stimuli. Further, we show that it is not only competent for quantitative screening of anti‐tumor drugs in a fashion of more closely resembling 3D in vivo TME, but capable of grading clinical human hepatic carcinoma specimens. These are crucial for many areas involved in biomedicine and biomedical engineering, such as mechanobiological characterization, drug screening and precision medicine.

## 2 Results

### 2.1 Fabrication and Experimental Setup of Electromechanical Coupling Chip Systems

**Figure 1:**
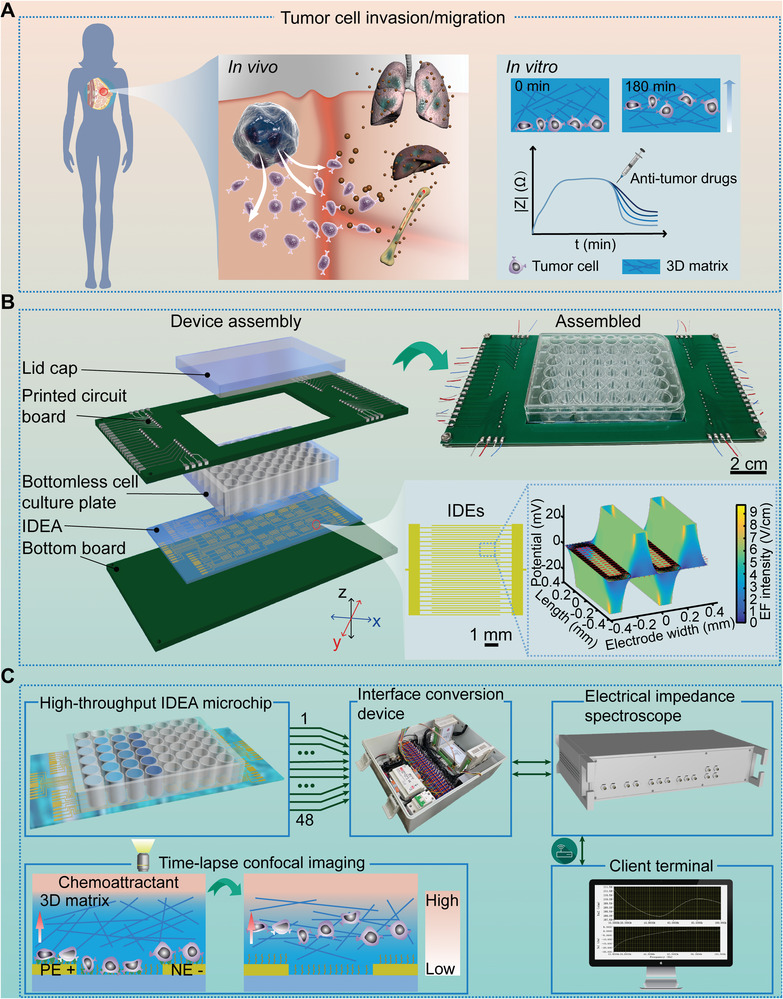
Fabrication and experimental setup of high‐throughput IDEA chip systems. A) Schematic representation of the metastasis process of cancer cells in vivo and in vitro. The in vitro migration and invasion in 3D extracellular matrices could be employed to test the efficacy of anti‐tumor drugs in principle. B) Schematic diagram of IDEA chip assembly. The designed printed circuit board (PCB) was connected to the fabricated ITO electrode arrays via a series of pogo pins in the chip. Then, they were further connected to the electrical impedance spectroscope for subsequent experiments. The chip consisted of 48 independent groups of ITO interdigitated electrodes (IDEs). The length and width of these electrodes were 7 mm and 100 µm, respectively, whereas the net distance between neighboring electrodes was designed as 100 µm. The images on the right below described the spatial distribution of electric field intensity on two pairs of interdigitated electrodes, when an alternating sinusoidal voltage of 30 mV was applied to detect the complex impedance signals. The colors displayed the absolute magnitude of the electric field intensity in V cm^−1^, whereas the arrows showed its relative magnitude and direction. C) Experimental system setup. The electromechanical coupling chip systems were composed of high‐throughput IDEA chips, interface conversion devices, electrical impedance spectroscopes and control and analysis systems. In experiments, the high‐throughput IDEA chip was placed in a tissue culture incubator for 3D cell culture and subsequent migration/invasion assay at 37 °C, 5% CO_2_, and 95% humidity. We sequentially measured the complex impedance signals in the range of 10–100 kHz within each chip chamber every certain period through the electrical impedance spectroscope. The signals were recorded and analyzed in a real‐time way. The images on the left below schematically described the upward collective cell migration assay in 3D extracellular matrices in response to a spatial chemoattractant gradient induced by cell culture medium with 10% FBS in the chip chambers.

The electromechanical coupling chip systems consisted of high‐throughput IDEA chips, interface conversion devices, electrical impedance spectroscopes and control and analysis systems (**Figure** **1**; Figure S1, Supporting Information). It allowed us to characterize spatiotemporal dynamics of 3D migration and invasion of cancer cells and quantitatively evaluate the influence of biomechanical and biochemical cues on these cells living 3D microenvironments through detecting complex impedance signals of the electromechanical coupling chip array (Figure 1A). In the work, the developed high‐throughput chip included 48 independent groups of ITO interdigitated electrodes (IDEs) on a glass substrate, which was prepared with a dry‐etching method based on a laser etching machine (Figure S3; Video S1, Supporting Information). The length and width of these electrodes were 7 mm and 100 µm, respectively, whereas the net distance between neighboring electrodes was designed as 100 µm. They were then fabricated by firmly bonding a bottomless cell culture plate onto the glass substrate with IDEs (Figure 1B, see Experimental Section for more details). Prior to 3D‐CIMA on the developed electromechanical coupling chip systems (Figure 1C; Figure S4, Supporting Information), we pipetted the cell suspension (2 × 10^5^ cells mL^−1^) into each individual chamber and cultured them at 37 °C in a humidified atmosphere with 5% CO_2_ for 24 h. Afterwards, they were starved for 12 h by treatments with 0.5% FBS‐contained medium. When the cells reached ~90% confluence, we added 0.2 mL of collagen I (Coll I) pre‐gel solution into each chamber and subsequently incubated it for gelation in an incubator for another 3 h. Next, the complete culture medium containing 10% FBS was pipetted into the chip chambers to create a chemoattractant gradient in the polymerized gel layers, which potentially cause collective cell migration in the chip chambers (Figure 1C).

**Figure 2:**
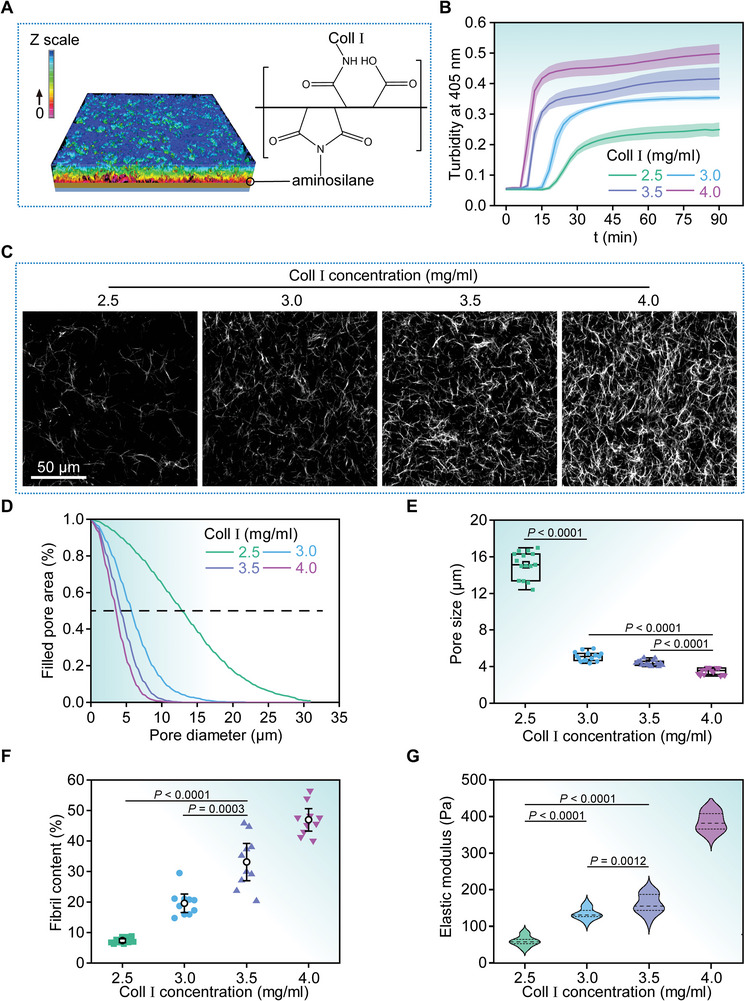
Topological analysis and mechanical characterization of Coll I matrices. A) The glass slides with IDEs were surface functionalized with poly(styrene‐*alt*‐maleic anhydride) (PSMA) to ensure covalent immobilization of Coll I matrices. B) Time‐dependent turbidity analysis describing the fibrillogenesis kinetics of Coll I matrices whose monomer concentrations were 2.5, 3.0, 3.5, and 4.0 mg mL^−1^, respectively. Data are shown from at least three independent experiments (*n* ≥ 3) with Mean ± SD. C) Optical slices showing topological microarchitectures of the Coll I matrices with different pre‐gel concentrations (i.e., 2.5, 3.0, 3.5, and 4.0 mg mL^−1^), which were obtained through a laser scanning confocal microscope (Nikon A1, Japan). D) Curves of filled pore area (%) versus pore diameter (µm) for the Coll I matrices with the pre‐gel monomer concentrations of 2.5, 3.0, 3.5, and 4.0 mg mL^−1^. These curves were obtained by quantifying the confocal images, as displayed in (C), based on a set of well‐established erosion algorithms (see Experimental Section for more details). The dashed line indicated the position where the filled pore areas were equal to 50%. E) Distributions of pore sizes acquired from the optical images as displayed in (C) using the set of erosion algorithms (see Experimental Section for more details). The boxplots also presented mean values and the corresponding standard deviations of the pore sizes, which essentially depended upon the monomer concentrations. F) Fibril contents of the Coll I hydrogels with different pre‐gel concentrations, which were obtained via an algorithm of automated topology analysis from the confocal image stacks (see Experimental Section for more details). Each data point represented the mean of 10 samples. G) Young's moduli of the Coll I hydrogel matrices whose monomer concentrations were 2.5, 3.0, 3.5, and 4.0 mg mL^−1^, respectively. These moduli were determined through a colloidal probe force spectroscope (see Experimental Section for more details). For each sample, at least 16 force‐distance curves at 3 locations were obtained from three independent experiments. The dashed lines represented the median and dotted lines denoted the quartiles.

Notice that the glass substrates with IDEs were beforehand surface‐functionalized with poly(styrene‐*alt*‐maleic anhydride) (PSMA) in order to covalently immobilize the Coll I hydrogel networks in situ (**Figure** **2**A, see Experimental Section for details). To mimic cellular microenvironments with specific mechanical cues, we prepared Coll I networks with different concentrations (2.5, 3.0, 3.5, and 4.0 mg mL^−1^) by accordingly diluting Coll I stock solution (rat tail Coll I, 8.91 mg mL^−1^ in 0.02 m acetic acid, Corning, New York, USA) in acetic acid (0.02 m, Sigma‐Aldrich, USA), which was then neutralized to pH 7.4 with 10× PBS, DMEM and 0.5 m NaOH (Sigma‐Aldrich, USA). To prevent premature fibril formation of the collagen monomers, we always kept and mixed all the ingredients on ice (4 °C) while avoiding the introduction of air bubbles into the solution. We carried out a turbidity analysis to characterize the kinetics of the Coll I fibril formation. To this end, we transferred the pre‐gel solution (100 µL) to a pre‐chilled (4 °C) 96‐well microplate (Corning, New York, USA) before polymerization, and loaded it into a pre‐warmed (37 °C) plate reader (ThermoFisher Scientific, USA) to quantify their optical densities at 405 nm for 90 min at 1 min intervals. At least three independent tests were performed in the current experiments (*n* ≥ 3). The turbidity analysis showed typical sigmoidal turbidity‐time curves with characteristic lag, growth and plateau phases (Figure 2B). It appeared that the fibrillogenic rate took on an increasing trend with the increase in Coll I monomer concentrations. Meanwhile, the lag phase shortened with increasing the Coll I concentration probably owing to the enhanced fibril nucleation capability. Considering the potential regulatory effects of microscopic geometry of the hydrogel networks on cellular adhesion, polarization, migration and invasion, we further performed a micro‐topological analysis to measure pore size and fibril morphology of the reconstituted collagen networks. In this context, the pre‐gel monomers were labeled with 5(6)‐TAMRA‐SE as red so that it could readily be visualized by a laser scanning confocal microscope (Nikon A1, Japan). Figure 2C presented representative topological microarchitectures of the Coll I matrices with different pre‐gel concentrations. It turned out that the hydrogel networks became much denser with increasing the concentrations while the fibril diameters were essentially independent of the Coll I concentrations (Figure 2C). Subsequently, we implemented a quantitative analysis based on a set of home‐built Matlab codes (see Experimental Section for details), which indicated that the pore sizes were ~15, 5, 4, and 3 µm for the hydrogel networks whose monomer concentrations were 2.5, 3.0, 3.5, and 4.0 mg mL^−1^ (Figure 2D−F), respectively. The sizes were very similar to that of collagen matrices in the interstitial tissues.^[^ ^33^ ^]^ Besides, we quantified the Young's moduli of the Coll I matrices with diverse monomer concentrations using a colloidal probe force spectroscope (see Experimental Section for details). They were 60.2, 134.6, 161.6, and 385.6 Pa for the hydrogel networks whose monomer concentrations were 2.5, 3.0, 3.5, and 4.0 mg mL^−1^, respectively (Figure 2G), which implied that a decrease in the pore size indeed led to a nonlinear increase in their Young's moduli. In essence, the specific Young's moduli exhibited by the fabricated collagen matrices were basically within the range of physiological stiffness of human tissues in vivo. The engineered collagen matrices were designed for in vitro cellular analysis that mimicked the complex in vivo 3D cellular environments.

### 2.2 Spatiotemporal Evolution of Complex Impedance Spectra Induced by 3D Migrating/Invading Cells in the Chip Systems

**Figure 3:**
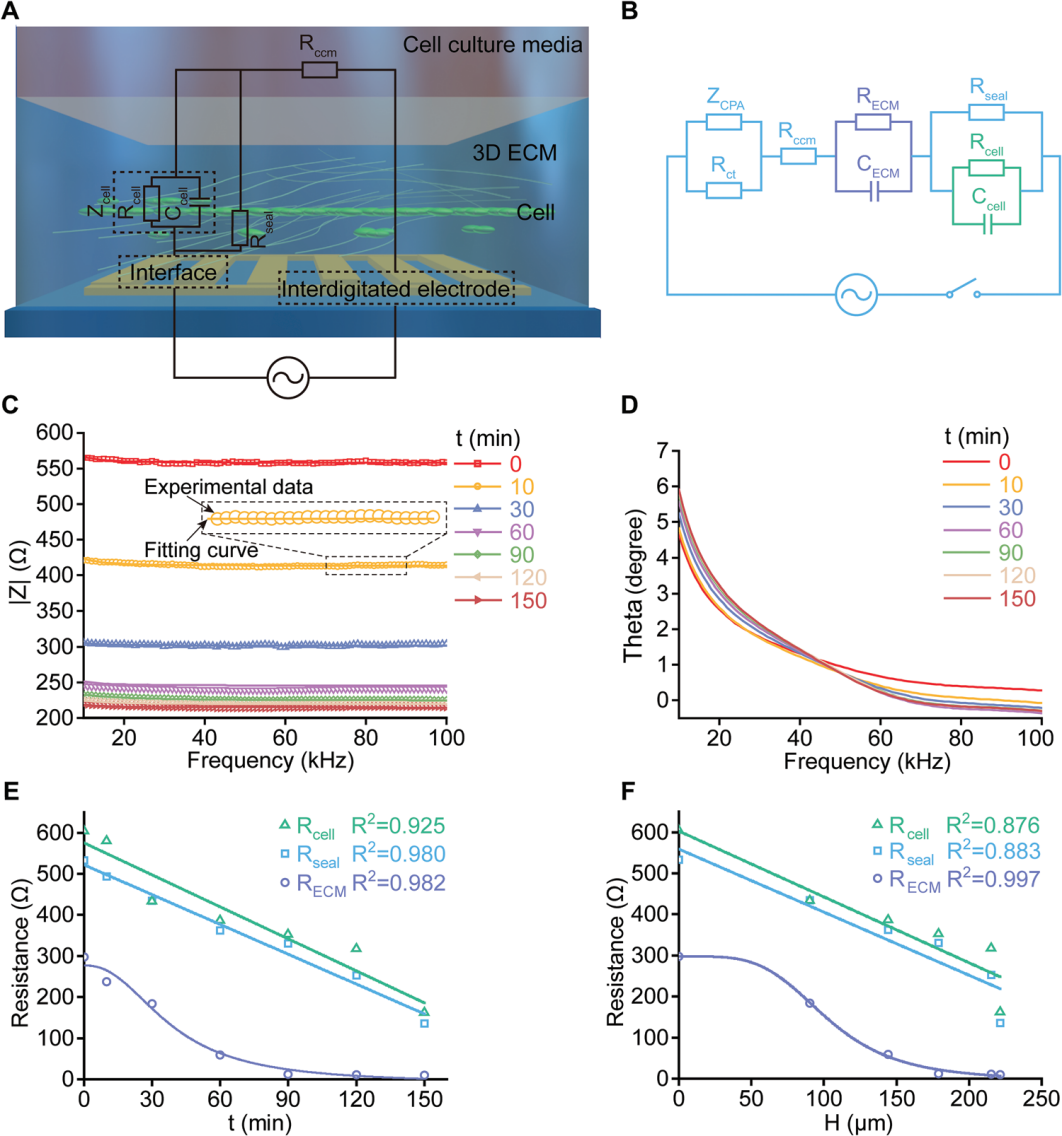
Spatiotemporal changes in complex impedance signals in response to 3D cell migration and invasion in the chip systems. A,B) An equivalent circuit model consisting of a series of electrical elements to characterize the complex impedance responses. In this model, *Z*_CPA_ denoted a constant phase angle impedance as defined in Equation (1); *R*_ct_ was a charge transfer resistance; *R*_ccm_ stood for the resistance of cell culture media; *R*_ECM_ and *C*_ECM_ indicated the equivalent resistance and capacitance of the 3D collagen matrices, respectively; *R*_seal_ denoted the seal resistance associated with the potential current shunting induced by the distances between the migrating/invading cells and the interdigitated electrode array as well as the extent of cell coverage on IDEs; *R*_cell_ and *C*_cell_ were the equivalent resistance and capacitance of the migrating/invading cells in a specific chip chamber, respectively. C) The magnitudes of representative complex impedance spectra (i.e., |*Z*(*ω*, **p**)|) in the range of 10–100 kHz measured at the time points of 0, 10, 30, 60, 90, 120, and 150 min after 3D migration/invasion of MDA‐MB‐231 cells cultured in 2.5 mg mL^−1^ Coll I matrix, respectively. The discrete data points represented the actually measured results while the solid curves denoted the corresponding fitting results based on the equivalent circuit model. D) The corresponding phase characteristics of the complex impedance spectra (i.e.,*Arg*[*Z*(*ω*, **p**)]). E,F) The changes in *R*_seal_, *R*_cell_, and *R*_ECM_ with time and average distance these cells moved away from the underlying IDEs, respectively. The discrete data were obtained by fitting the measured complex impedance spectra with the proposed equivalent circuit model, whereas the solid curves were the corresponding data fitting results.

In the 3D cell invasion/migration assay, the changes in complex impedance spectra of the electromechanical coupling chip systems intrinsically reflect the spatiotemporal dynamics of 3D invasion/migration of the cells away from the interdigitated electrode array (**Figure** **3**A).^[^ ^32^, ^34^, ^35^ ^]^ To characterize the time‐related process of cell invasion/migration in the 3D extracellular matrices, we introduced an equivalent circuit model including a series of key electrical elements (Figure 3A,B). In the model, the resistance of cell culture media was denoted as *R*_ccm_, which was essentially a function of the total ion concentration in the media and ambient temperature,^[^ ^10^, ^36^ ^]^ whereas the interfacial effect between the interdigitated electrodes and the media that behaved as electrolytes, was modeled as a constant phase angle impedance *Z*_CPA_ in parallel with a charge transfer resistance *R*_ct_, which characterized the interface capacitance impedance and the capability of the electrodes to participate in exchange current reactions, respectively.^[^ ^37^, ^38^ ^]^ Specifically, the constant phase angle impedance could be quantitatively expressed as

(1) $$Z_{\mathrm{CPA}}\left(\omega ,Q,n\right)=\frac{1}{Q{\left(j\omega \right)}^{n}}$$

where *Q* denoted the magnitude of *Z*_CPA_, *n* was a constant with 0 ≤ *n* ≤ 1 that characterized the inhomogeneities of the electrode surfaces, *ω* was the angular frequency, and *j* stood for the imaginary unit.^[^ ^37^, ^38^ ^]^ The complex impedance of the migration/invasion cells could be simplified as an equivalent capacitance *C*_cell_ in parallel with a resistance *R*_cell_.^[^ ^36^, ^39^, ^40^ ^]^ Subsequently, a seal resistance, that is, *R*_seal_, was introduced to account for the potential current shunting caused by the distances between the migrating/invading cells and the interdigitated electrode array, and the degree of cell coverage on the electrodes.^[^ ^41^ ^]^ Next, the resistance of the 3D extracellular collagen matrices was represented as an equivalent capacitance *C*_ECM_ in parallel with a resistance *R*_ECM_ in the circuit model. In this context, the complex impedance of the electromechanical coupling chip systems could be equivalently expressed as

(2) $$\begin{array}{ccc} Z\left(\omega ,p\right) & = & \frac{R_{\mathrm{ct}}Z_{\mathrm{CPA}}\left(Q,n\right)}{R_{\mathrm{ct}}+Z_{\mathrm{CPA}}\left(Q,n\right)}+R_{\mathrm{ccm}}+\frac{R_{\mathrm{ECM}}}{j\omega C_{\mathrm{ECM}}R_{\mathrm{ECM}}+1}\\ & & +\;\frac{R_{\mathrm{seal}}R_{\mathrm{cell}}}{j\omega C_{\mathrm{cell}}R_{\mathrm{seal}}R_{\mathrm{cell}}+R_{\mathrm{seal}}+R_{\mathrm{cell}}} \end{array}$$

where **p** = [*R*_ccm_,*R*_ECM_,*C*_ECM_,*R*_seal_,*R*_cell_,*C*_cell_,*R*_ct_,*Q*, *n*]^*T*^. Afterwards, we defined a cost function *e*(**p**) in the least‐squares sense,^[^ ^42^ ^]^ namely,

(3) $$e\left(p\right)=\sum_{i=1}^{N_{T}}{\left[Z_{i}-Z\left(\omega_{i},p\right)\right]}^{2}$$

where *N*_T_ was referred to as the total number of sampling points in experiment, *Z*_i_ denoted the measured complex impedance value at the frequency of *ω*_i_ with 1 ≤ *i* ≤ *N*_T_. Minimizing the cost function with respect to **p** required ∂[*e*(**p**)]/∂**p** = 0, which could readily be solved in the way of numerical iterations in Matlab (Mathworks).

In the chip systems, the complex impedance spectra corresponding to each chip chamber could be collected automatically with the aid of an HF2IS Impedance Spectroscope (Zurich Instruments, Switzerland). The amplitude of the sinusoidal voltage applied to each IDE array was 30 mV while the frequency sweep range was from 10 to 100 kHz. Notice that the impedance signals within the specified range were generally more sensitive to the 3D motion of cells (Figure 3C,D). In this way, we could detect the spatiotemporal dynamics of complex impedance spectra in a high‐throughput fashion and therefore investigate collective migration/invasion of cells in the collagen matrices with diverse geometric and mechanical properties. Figure 3C showed the magnitudes of the complex impedance spectra in the range of ≈10–100 kHz acquired at the time points of 0, 10, 30, 60, 90, 120, and 150 min after 3D migration/invasion of MDA‐MB‐231 cells, whereas Figure 3D presented the corresponding phase characteristics of the impedance spectra. Further, we fitted the experimental data with Equations (2) and (3), as shown by these solid curves in Figure 3C. It could be observed that the fitting curves were in good agreement with the experimental data, suggesting that the equivalent circuit model was capable of characterizing the complex impedance response of the chip systems. Of note, it appeared that both of the cell resistance *R*_cell_ and the seal resistance *R*_seal_ were linearly related to the duration of the 3D cell migration/invasion and the average distance these cells moved away from the underlying IDEs, respectively (Figure 3E,F), although the other parameters took on a nonlinear dependence in the process (Figure 3E,F; Figures S6 and S15 and Tables S2–S5, Supporting Information). The linear dependence of *R*_cell_ and *R*_seal_ on the migrating/invading time and distance indicated that the measured complex impedance signals could be employed as a stable and reliable index to capture the spatiotemporal dynamics of 3D cell migration and invasion in the developed electromechanical coupling chip systems.

### 2.3 Quantitative Comparisons with Experimental Data Presented by Laser Scanning Confocal Microscopy

**Figure 4:**
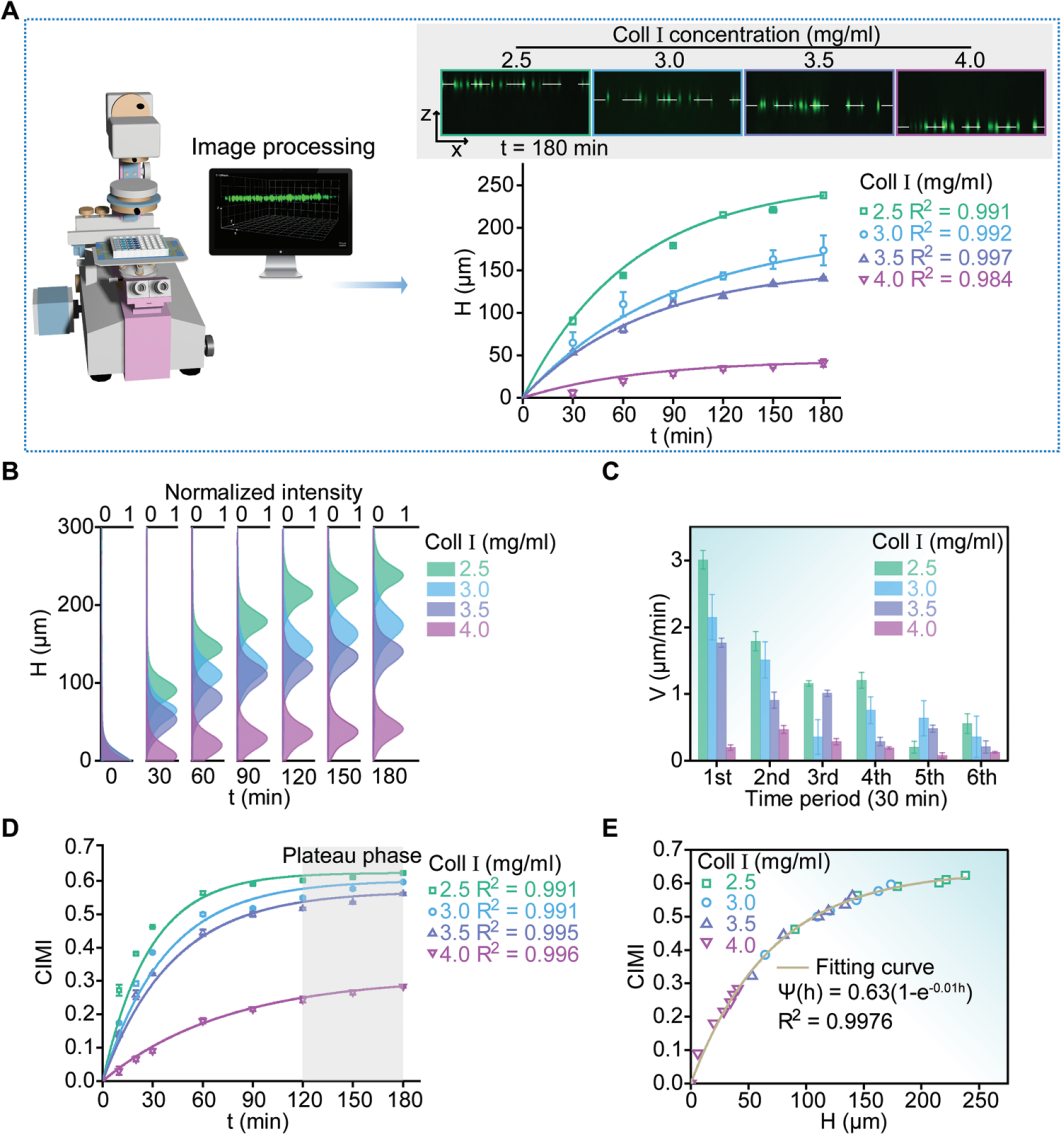
Quantitative comparisons between spatiotemporal processes of cell invasion/migration in 3D matrices and the corresponding variations in complex impedance signals. A) Upward invasion/migration processes of MDA‐MB‐231 cells grown in 3D collagen matrices in response to a chemoattractant gradient. The cells were beforehand labeled as green for visualization with Cell Tracker Green CMFDA. The invading/migrating processes were recorded with a laser scanning confocal microscope (Nikon A1, Japan). The four insets on the upper right presented spatial positions of the cells after 180 min of upward invasion/migration in the collagen matrices whose monomer concentrations were 2.5, 3.0, 3.5, and 4.0 mg mL^−1^, respectively. The graph on the lower right showed the distances the cells living in the four Coll I matrices migrated/invaded upward from the electrode surface in 180 min. The time‐related distances were quantified every 30 min with the laser scanning confocal microscope. The solid curves were the corresponding fitting results where all R^2^ were greater than 0.98. B) Spatiotemporal distributions of the upward invading/migrating cells that were labeled as green with Cell Tracker Green CMFDA in the 3D matrices. The Gaussian‐like spatial distributions displayed 3D collective cell migrating/invading dynamics of the MDA‐MB‐231 cells at different time points. The spatial distributions of migrating/invading cells were obtained via quantifying their green fluorescent intensities in the acquired 3D volumetric images using a set of self‐written Matlab codes. C) Average invasion/migration velocities per half hour of the MDA‐MB‐231 cells grown in 3D collagen matrices in response to the specific chemoattractant gradient. Notice that the average velocities in first, second, third, fourth, fifth, and sixth time periods denoted those over 0–30, 30–60, 60–90, 90–120, 120–150, and ≈150–180 min, respectively, after upward invasion/migration of the cells. D) Variations in cell invasion/migration index (CIMI) with time, which were obtained through 3D‐CIMA. The discrete data indicated the experimental results measured via 3D‐CIMA whereas the solid curves represented the fitting results based on the formula of $\Psi \left(t\right)=\Psi_{0}\left(1-e^{-t/\tau_{\Psi }}\right)$. Data were shown as Mean ± SD (*n* = 3). E) Quantitative comparison between the invasion/migration distances away from IDEs and the corresponding CIMIs measured by 3D‐CIMA. These discrete points denoted the experimental data while the solid curve was the corresponding fitting result with $\Psi \left(h\right)=\Psi_{h}\left(1-e^{-h/h_{\Psi }}\right)$ where Ψ_h_ = 0.63, $h_{\Psi }=100$, and *R* ^2^ = 0.9976.

To minimize the effect of cell density, morphology and adhesion strength on IDEs in the chip chambers, we defined a cell invasion/migration index (CIMI) to quantitatively characterize the spatiotemporal dynamical process of cell invasion and migration in the 3D collagen matrices by normalizing the impedance data

(4) $$\Psi =\arg\max_{\omega }\left|\frac{\left|Z_{t}\left(\omega ,p\right)\right|-\left|Z_{0}\left(\omega ,p\right)\right|}{\left|Z_{0}\left(\omega ,p\right)\right|}\right|$$

where *Z*_0_(*ω*,**p**) and *Z*_t_(*ω*,**p**) represented the complex impedance signals sampled at the time points of 0 and *t*, respectively. Subsequently, we detected the 3D cell migration/invasion processes with a laser scanning confocal microscope (**Figure** **4**A). In the presence of a vertical chemoattractant gradient in the 3D collagen matrices, the green fluorescence‐labeled cells initially attached to IDEs could move upward spontaneously (Figure 4A). The rate of invasion/migration decreased dramatically as the monomer concentration of the collagen matrix was increased from 2.5 to 4.0 mg mL^−1^ (Figure 4A). This might be attributed to the inhibition of directional cell invasion/migration by the surrounding collagen matrices with densely crosslinked densities. As such, it turned out that the spatiotemporal dynamics of the upward invading/migrating cells could be well characterized with the formula of *H*(*t*) = *H*_0_(1 − *e* ^−*t*/*τ*^) with R^2^ > 0.98, where *t* represented the invading/migrating duration, *H*(*t*) denoted the vertical invading/migrating distance of the cells in the 3D matrices with time, *τ* = 100 was a characteristic time and *H*_0_ was a coefficient that depended on the monomer concentration of the 3D collagen matrices (Figure 4A). Besides, we noticed that the coefficient *H*_0_ was roughly inversely proportional to the monomer concentration used for collagen polymerization (Figure S7A, Supporting Information). A quantitative analysis further indicated that these MDA‐MB‐231 cells underwent collective cell invasion/migration in the 3D collagen matrices with different monomer concentrations (Figure 4B and Video S2, Supporting Information). In all the groups, the average invasion/migration velocities gradually decayed over time (Figure 4C), possibly due to a decrease in the chemoattractant gradient as the cells moved away from the underlying IDEs. On the other hand, we found CIMI increased rapidly at the initial invasion/migration phases, but basically reached a plateau after 120 min (Figure 4D), which implied that CIMI was more sensitive to the initial phase of cell invasion/migration. Finally, we made a comparative analysis of CIMI and the corresponding invasion/migration distance of the cells. In the current experimental configuration, we observed that, regardless of the monomer concentrations of the 3D collagen matrices, the quantitative relationship between CIMI and the invasion/migration distance might be well characterized by the formula of $\Psi \left(h\right)=\Psi_{h}\left(1-e^{-h/h_{\Psi }}\right)$ in which Ψ_h_ = 0.63, $h_{\Psi }=100$ and *R* ^2^ = 0.9976(Figure 4E). This suggested that CIMI obtained from 3D‐CIMA allowed us to characterize spatiotemporal dynamics of 3D invasion/migration in a quantitative fashion.

### 2.4 Quantitative Evaluation of Drug Efficacy Based on 3D‐CIMA

**Figure 5:**
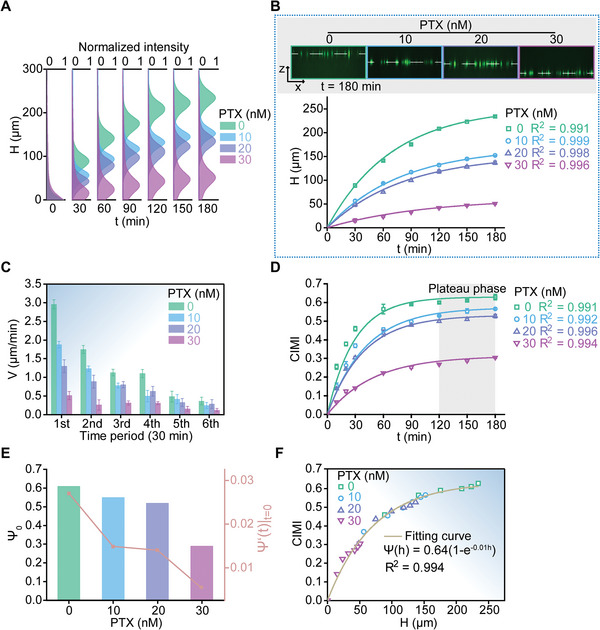
Efficacy evaluation of anti‐tumor drugs based on 3D‐CIMA. Prior to the experiments, MDA‐MB‐231 cells encapsulated in 2.5 mg mL^−1^ collagen matrix were pretreated with different concentrations (10, 20, and 30 nm) of paclitaxel (PTX, Sigma‐Aldrich, USA), respectively. A) Spatiotemporal distributions of 3D invading/migrating cells that were beforehand labeled as green with Cell Tracker Green CMFDA. The Gaussian‐like spatial distributions exhibited 3D collective cell invading/migrating behavior at different time points. The spatiotemporal dynamics were detected with a laser scanning confocal microscope (Nikon A1, Japan) in a real‐time way, where a series of 3D volumetric images were taken to record the 3D invading/migrating processes of the green fluorescence‐labeled cells cultured in the collagen matrix. B) Inhibition of PTX on invasion/migration of the MDA‐MB‐231 cells in the collagen matrix, which was quantified with the conventional confocal fluorescence imaging method. In the experiments, spatiotemporal dynamics of upward invasion/migration of the MDA‐MB‐231 cells were recorded with the laser scanning confocal microscope. The invasion/migration distances away from IDEs were quantified from the acquired 3D volumetric images using a set of self‐written Matlab codes. The solid curves were the corresponding fitting results based on the formula of $\Psi \left(h\right)=\Psi_{h}\left(1-e^{-h/h_{\Psi }}\right)$ with $h_{\Psi }=100$. The four insets on the upper part showed spatial positions of the green fluorescence‐labeled cells after 180 min of upward invasion/migration in the collagen matrix, where the PTX concentrations were 0, 10, 20, and 30 nm, respectively. Note that the group labeled as “0 nm” was the control group in the experiments. C) Average invasion/migration velocities per half hour of the cells treated with PTX of 0, 10, 20, and 30 nm, respectively. Notice that the average velocities in first, second, third, fourth, fifth, and sixth time periods denoted those over 0–30, 30–60, 60–90, 90–120, 120–150, and 150–180 min, respectively, after upward invasion/migration of the cells in the 3D collagen matrix. D) Time‐dependent CIMI data for the cells treated with PTX of 0, 10, 20, and 30 nm, respectively. Data were shown as Mean ± SD (*n* = 3). The solid curves were the corresponding fitting results on the basis of the formula of $\Psi \left(t\right)=\Psi_{0}\left(1-e^{-t/\tau_{\Psi }}\right)$. E) Steady‐state CIMIs (i.e., $\Psi_{0}=\lim_{t\to +\infty }\left[\Psi (t)\right]$) and their derivatives with respect to time at *t* = 0 (i.e., [*d*Ψ(*t*)/*dt*]_*t* =_ )=0 for the 3D invading/migrating cells treated with PTX of 0, 10, 20, and 30 nm. F) Quantitative comparison between the invasion/migration distances away from IDEs and the corresponding CIMIs quantified by 3D‐CIMA. These discrete points were the experimental data whereas the solid curve was the fitting result with $\Psi \left(h\right)=\Psi_{h}\left(1-e^{-h/h_{\Psi }}\right)$ where Ψ_h_ = 0.64, $h_{\Psi }=100$ and *R* ^2^ = 0.994.

The developed 3D‐CIMA was adapted to quantitively evaluate the efficacy of anti‐tumor drugs in a nondestructive way. In clinic, paclitaxel was a chemotherapeutic agent widely used to treat many different types of cancers including breast cancer because it suppressed dynamic instability of microtubules when administered at clinically relevant concentrations in the nanomolar range.^[^ ^43^ ^]^ At relatively low concentrations, paclitaxel no longer affected cell viability, but was still effective in reducing cell invasiveness and motility (Figure S8, Supporting Information). In the experiments, we first treated MDA‐MB‐231 cells encapsulated in 2.5 mg mL^−1^ collagen matrix with different concentrations (10, 20, and 30 nm) of paclitaxel (PTX, Sigma‐Aldrich, USA), respectively. To facilitate the tracking of the 3D invasion/migration processes with a laser scanning confocal microscope, we also simultaneously labeled the cells as green with Cell Tracker Green CMFDA in advance. Then, we detected the collective invasion/migration process of the PTX‐treated cells in the 3D collagen matrix. With the laser scanning confocal microscope, we monitored spatiotemporal dynamics of the invading/migrating cells pre‐stimulated with different concentrations of PTX (**Figure** **5**A,B). As the concentration of PTX increased, the 3D invasion/migration ability of the cancer cells gradually decreased. In this context, the time‐dependent upward invasion/migration distances could also be well characterized with the expression of $\Psi \left(h\right)=\Psi_{h}\left(1-e^{-h/h_{\Psi }}\right)$ with $h_{\Psi }=100$ and *R* ^2^ > 0.99 (Figure 5B; Figure S9B, Supporting Information). The invasion/migration velocities decreased significantly with increasing the PTX concentration (Figure 5C; Figure S9C, Supporting Information). These facts implied that the antineoplastic drug could effectively inhibit the 3D invasion and migration of the breast cancer cells. At the same time, we obtained the corresponding CIMIs of the 3D invading/migrating cells treated with different concentrations of PTX based on the developed 3D‐CIMA, which also satisfied the formula of $\Psi \left(t\right)=\Psi_{0}\left(1-e^{-t/\tau_{\Psi }}\right)$ approximately (Figure 5D; Figure S9D, Supporting Information). This suggested that CIMI increased rapidly during the initial invasion/migration phase and gradually arrived at a plateau. It was obvious that the steady‐state CIMI (i.e., $\Psi_{0}=\lim_{t\to +\infty }\left[\Psi (t)\right]$) and their derivatives with respect to time at the initial moment (i.e., [*d*Ψ(*t*)/*dt*]_*t* = 0_) were essentially directly associated with the PTX concentration adopted in these experiments (Figure 5E). The correlation between the measured CIMIs and the corresponding invasion/migration distances away from IDEs could still be quantitatively described by the formula of $\Psi \left(h\right)=\Psi_{h}\left(1-e^{-h/h_{\Psi }}\right)$ where Ψ_h_ = 0.64, $h_{\Psi }=100$ and *R* ^2^ = 0.994 (Figure 5F).

**Figure 6:**
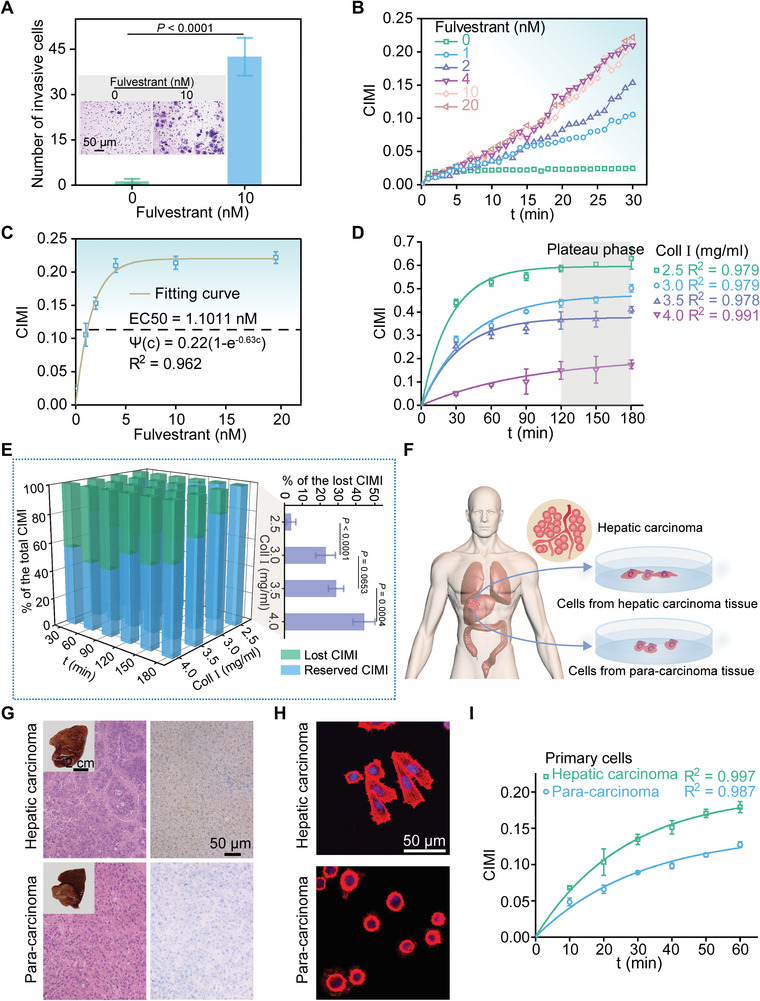
3D invasion/migration‐based drug efficacy evaluation and quantitative characterization of invasion/migration capabilities of primary cells from hepatic carcinoma and para‐carcinoma tissues. A) Statistical results of the number of invasive cells (MCF‐7 cells) based on a classical transwell assay, which displayed the regulatory effect of fulvestrant on 3D invasion of the cells (*n* = 3). This pair of insets showed typical spatial distributions of invading cells without/with fulvestrant stimulation in the transwell assay. B) Time‐related CIMIs measured by the developed 3D‐CIMA in a real‐time fashion for the MCF‐7 cells cultured in the collagen matrix of 2.5 mg mL^−1^, which were beforehand stimulated with fulvestrant of 0, 1, 2, 4, 10, and 20 nM, respectively. Note that the group labeled as “0” represented the control group in the experiments. The showed CIMI data had been normalized by those obtained in the control group. C) CIMI as a function of fulvestrant concentration. This discrete data (Mean ± SD, *n* = 3) indicated the experimental results of CIMIs obtained by 3D‐CIMA whereas the curve was the fitting results with Ψ(*c*) = 0.22(1 − *e* ^−0.63*c*^) (*R* ^2^ = 0.962), in which *c* denoted the concentration of fulvestrant in nM. The half maximal effective concentration (EC50) was found to be 1.1011 nM for the cells cultured in the collagen matrix of 2.5 mg mL^−1^. D) Time‐dependent CIMI data for MDA‐MB‐231 cells cultured in the collagen matrices of 2.5, 3.0, 3.5, and 4.0 mg mL^−1^, respectively, in the presence of GM6001 (Ilomastat) of 20 µM, which was a potent and broad spectrum matrix metalloprotease (MMP) inhibitor. Data were shown as Mean ± SD (*n* = 3). The solid curves were the corresponding fitting results based on the formula of $\Psi \left(t\right)=\Psi_{0}\left(1-e^{-t/\tau_{\Psi }}\right)$ with Ψ_0_ = 0.23, 0.38, 0.47, and 0.59 for the collagen matrices of 2.5, 3.0, 3.5, and 4.0 mg mL^−1^, respectively. E) Comparisons between lost and reserved CIMIs for the MDA‐MB‐231 cells treated with/without GM6001 (Ilomastat) of 20 µM, in the matrices of 2.5, 3.0, 3.5, and 4.0 mg mL^−1^. F) Schematic depiction of primary hepatic carcinoma cells and paracarcinoma cells collected from a liver cancer patient. G) Hematoxylin and eosin (H&E) and alpha fetoprotein (AFP) staining of the primary hepatic carcinoma specimens. These two insets were the photographs of the hepatic carcinoma and paracancerous tissue specimens. H) Immunofluorescence images of primary hepatic carcinoma cells and paracarcinoma cells, where cell nuclei and cytoskeletons were labeled as blue and red, respectively. I) Time‐dependent CIMI data (Mean ± SD, *n* = 3) for the primary hepatic carcinoma cells and paracarcinoma cells cultured in the Coll I matrix of 2.5 mg mL^−1^. The solid curves were the fitting results based on the formula of $\Psi \left(t\right)=\Psi_{0}\left(1-e^{-t/\tau_{\Psi }}\right)$ with Ψ_0_= 0.197 and $\tau_{\Psi }$= 26.316 for the primary hepatic carcinoma cells, and Ψ_0_ = 0.143 and $\tau_{\Psi }$ = 31.250 for the paracarcinoma cells.

We subsequently evaluated 3D invasive and metastatic ability of breast cancer cells (MCF‐7) regulated by oestrogen receptor alpha (ER*α*). In clinic, ER*α* was used as another key target for endocrine therapy in the ER*α*‐positive breast cancers, which accounted for ~70% of all breast cancers.^[^ ^44^, ^45^ ^]^ As a transcriptional promoter of vinculin that was a cytoskeletal protein associated with linkage of transmembrane integrins to intracellular actin cytoskeletons, ER*α* expressed in the MCF‐7 cells might be downregulated by fulvestrant (Figure S10A,B, Supporting Information), which could in turn promote focal adhesion turnover and thus increase their invasive and metastatic ability,^[^ ^46^ ^]^ as demonstrated by our transwell assay (**Figure** **6**A). With the proposed 3D‐CIMA, we revealed that the time‐dependent CIMI data were directly related to the fulvestrant concentration (Figure 6B). The relative CIMI data rose rapidly and eventually plateaued as the fulvestrant concentration increased to 20 nM (Figure 6C). In this way, we estimated that the half maximal effective concentration (EC50) of fulvestrant was ≈1.1011 nMm for the cells grown in the collagen matrix of 2.5 mg mL^−1^, whereas it was ≈1.8318 nM for the cells in the matrix of 3.0 mg mL^−1^ (Figure S20, Supporting Information).

As another example, we quantitatively characterized the invasion/migration capability of cells regulated by matrix metalloproteinases (MMPs). The cell‐secreted MMPs were capable of degrading ECM proteins, thereby modulating local mechanical properties of the collagen matrix by altering matrix fiber density and matrix porosity. To this end, we encapsulated MDA‐MB‐231 cells into the chip systems and then monitored their upward invasion/migration processes in the collagen matrices. Meantime, we employed GM6001 (Ilomastat) to treat the cells, which was a potent and broad spectrum MMP inhibitor. The spatiotemporal dynamics were detected with a laser scanning confocal microscope (Nikon A1, Japan) in a real‐time way (Figure S10C, Supporting Information). It could be found that the invasion/migration velocities of the cells treated with 20 µM GM6001 were effectively inhibited in comparison with those arising in the control groups (Figure S10D, Supporting Information), which implied that the MMP‐induced matrix biodegradation could exert an important impact on 3D cell invasion/migration. Also, we obtained the time‐dependent CIMI data for the cells cultured in the collagen matrices of 2.5, 3.0, 3.5, and 4.0 mg mL^−1^, respectively, in the presence of 20 µM GM6001 (Figure 6D), where the measured data (Mean ± SD, *n* = 3) could also be well characterized with the formula of $\Psi \left(t\right)=\Psi_{0}\left(1-e^{-t/\tau_{\Psi }}\right)$ with Ψ_0_= 0.23, 0.38, 0.47, and 0.59 for the collagen matrices of 2.5, 3.0, 3.5, and 4.0 mg mL^−1^, respectively. The presence of GM6001 might lead to a significant loss of CIMI due to its marked inhibitory effect on the secretion of MMPs (Figure 6E). Finally, we collected primary hepatic carcinoma cells and paracarcinoma cells from a liver cancer patient to test whether the developed 3D‐CIMA could distinguish their differences in 3D invasive and metastatic abilities (Figure 6F–H). The upward invasion/migration process of the two types of cells cultured in 2.5 mg mL^−1^ collagen matrix were recorded with 3D‐CIMA, as displayed in Figure 6I where the measured CIMI data were presented as Mean ± SD (*n* = 3). The solid curves showed the fitting results based on the expression of $\Psi \left(t\right)=\Psi_{0}\left(1-e^{-t/\tau_{\Psi }}\right)$ with Ψ_0_= 0.197 and $\tau_{\Psi }$= 26.316 for the primary hepatic carcinoma cells, and Ψ_0_= 0.143 and $\tau_{\Psi }$= 31.250 for the paracarcinoma cells. These data implied that it could, in principle, assist us in grading clinical human liver cancer specimens.

## 3 Discussion and Conclusion

Cell invasion and migration are not only implicated in a series of complicated biochemical/biophysical interactions with ECM, but also trigger a cascade of signal transduction pathways. Quantitative characterization of cell invasion/migration can uncover abundant information about cellular functions and behaviors, therefore providing crucial clues for studies on mechanobiology and drug screening. Although traditional 2D cell‐based assays, for example, wound healing/scratch assay, are simple and widely employed to detect the movement of cells adhered on 2D flat substrates under various controlled conditions, the 2D cell‐based techniques are normally difficult to reproduce the 3D migration and invasion process of their in vivo cellular counterparts and functional responses under similar stimuli.^[^ ^5^, ^6^, ^8^, ^11^, ^12^ ^]^ This inherent limitation makes the conventional 2D cell‐based assays sometimes less accurate, and even produces misleading or false positive results.^[^ ^47^, ^48^ ^]^ Some novel drug candidates, for instance, exhibit excellent efficacy in the process of 2D cell‐based in vitro assays, but they may be less effective in vivo owing to the intrinsic distinction between in vivo and in vitro microenvironments.^[^ ^31^, ^49^ ^]^ Another disadvantage of the 2D cell‐based assays lies in that they usually ignore the regulatory effect of biophysical and biomechanical aspects of local ECM microenvironments on cell invasion and migration. Some electric cell‐substrate impedance sensing (ECIS)‐based techniques, for example, have been well developed for dynamic, non‐invasive and label‐free characterization of cell growth/proliferation and viability in the past decades,^[^ ^50^, ^51^, ^52^ ^]^ but they are difficult to characterize spatiotemporal dynamics of 3D invasion/migration regulated by ECM microenvironments with various biophysical/biomechanical properties. As a matter of fact, biophysical/biomechanical cues of ECM, such as stiffness, pore size and fiber density of ECM, play a pivotal role in modulating directional migration and invasion, and can even lead to switching of cell migration modes.^[^ ^2^, ^5^ ^]^

In the developed electromechanical coupling chip systems, we introduced 3D topological networks of Coll I matrices with adjusted biophysical/biomechanical properties to simulate the microenvironment of 3D cell invasion and migration in vivo, considering that the collagen matrices shared specific similarities with ECM in vivo in terms of matrix compositions, geometric features, biophysical/biomechanical properties.^[^ ^12^, ^13^, ^14^, ^15^ ^]^ In principle, it should also be feasible to use other types of matrix materials,^[^ ^12^, ^14^, ^22^, ^28^, ^53^, ^54^ ^]^ such as matrigel, synthetic matrix or biocompatible hydrogels with controllable biophysical, biochemical and biomechanical properties, in the current chip systems. This is crucial for some quantitative investigations related to mechanobiology and drug screening, because, to our knowledge, the existing cell‐based assays remain difficult to characterize the regulatory effect of biophysical/biomechanical aspects of ECM microenvironments on 3D cell invasion and migration. The developed 3D‐CIMA allows us to collect the CIMI data in the chip systems and hence evaluate spatiotemporal dynamics of cell invasion and migration through 3D ECM environments. The changes in the CIMI signals over time essentially reflect the dynamics of 3D invasion/migration of cells in response to external stimuli. After optical calibration with a laser scanning confocal microscope, the CIMI signals can be employed to more accurately characterize the spatiotemporal process of 3D collective cell invasion/migration induced by external chemokines in a real‐time, label‐free, nondestructive, and high‐throughput fashion. In the future, it is expected to achieve intelligent detection and characterization of 3D invasion and migration in the chip systems with the aid of some well‐established machine learning algorithms,^[^ ^55^, ^56^ ^]^ which is indispensable for many fields associated with biomedicine and biomedical engineering like mechanobiological analysis, drug screening and precision medicine in clinic.

## 4 Experimental Section

### Fabrication of High‐Throughput Interdigitated Electrode Array Chips

In this work, the high‐throughput IDEA chip consisted of a bottomless cell culture plate and a patterned indium tin oxide (ITO) plated glass slide. The length and width of a single electrode were designed as 7 mm and 100 µm, respectively, whereas the gap between adjacent electrodes was set as 100 µm, as illustrated in Figure S3, Supporting Information. IDEA was fabricated with a dry‐etching process through a laser etching machine whose laser wavelength and frequency were 1064 nm and 40 kHz, respectively. The corresponding marking speed was set as 1000 mm s^−1^ in experiment. Subsequently, a bottomless cell culture plate was glued onto the prepared IDEA substrate using the prepolymer of polydimethylsiloxane (PDMS, Sylgard 184, Dow Corning) with a weight ratio of 20:1 (base/curing agent) at 65 °C for 2 h.

Also, the glass slides with IDEs were beforehand surface‐functionalized with poly(styrene‐*alt*‐maleic anhydride) (PSMA) to ensure covalent immobilization of 3D Coll I networks on them, as described in some previous literature.^[^ ^53^ ^]^ In brief, the glass substrates were first pre‐cleaned with methanol (Thermo Fisher Scientific, USA) and amino‐salinated with 3‐aminopropyltrimethoxysilane (APTES, Sigma‐Aldrich, USA). Then, PSMA copolymer (MW 20 000–30 000 g mol^−1^, Sigma‐Aldrich, USA) solution in acetone (0.14 wt%) and tetrahydrofurane (Sigma‐Aldrich, USA) at a ratio of 1:2 were spin‐coated onto the substrates. The presence of anhydride groups during collagen fibril formation enabled the lysine side chains to bind to the covalent collagen surfaces.^[^ ^54^ ^]^

### 3D Cell Culture System Setup

The fabricated IDEA chips were pre‐cleaned with methanol (Thermo Fisher Scientific, USA) and amino‐salinated with 3‐aminopropyltrimethoxysilane (APTES, Sigma‐Aldrich, USA) solution (APTES in acetone at a volume ratio of 1:25). Then, poly(styrene‐*alt*‐maleic anhydride) (PSMA) copolymer (MW: 20 000–30 000 g mol^−1^, Sigma‐Aldrich, USA) solution in acetone (0.14 wt%) and tetrahydrofurane (Sigma‐Aldrich, USA) at a ratio of 1:2 were spin‐coated onto the chip substrate. The surface functionalized chips were sterilized with ultraviolet (UV) for 30 min prior to seeding cells.

For subsequent 3D cell culture, the cell suspension (2 × 10^5^ cells mL^−1^) was pipetted into each chip chamber and then cultured them in the complete culture medium containing 10% fetal bovine serum (FBS, Gibco, Life Technologies, USA) and 1% antibiotic (penicillin/streptomycin, Thermo Fisher Scientific, USA) for 24 h. Subsequently, they were treated with 0.5% FBS‐contained medium for starvation for another 12 h. All cell cultures were maintained at 37 °C in a humidified atmosphere with 5% CO_2_. For the anti‐tumor testing experiments, the cells were further pretreated with different concentrations of paclitaxel (PTX, Sigma‐Aldrich, USA) or fulvestrant (Sigma‐Aldrich, USA) for an additional 2 h. At the time point when the cells reached ~90% confluence, collagen I (Coll I) mixture was transferred into the chip chambers and incubated for gelation in a tissue culture incubator at 37 °C, 5% CO_2_ and 95% humidity for 3 h. Note that a relatively low cell density was likely to reduce the sensitivity of subsequent impedance measurements while an excessively high cell density might produce cell contact inhibition. Next, the pre‐warmed (37 °C) complete culture medium containing 10% FBS was added in the chip chambers, which could form a chemoattractant gradient along the thin layers of Coll I gels and thus induce upward collective cell migration in the chip chambers. In the experiments, it was recommended that the thickness of the Coll I hydrogel layers should not exceed 2000 µm because an increase in the layer thickness might affect the diffusion of chemokine FBS and other nutrients.

### Characterization of Microstructures Based on Scanning Electron Microscopy

Microstructures of 3D collagen gels were visualized by a TESCAN MAIA3 ultra high‐resolution field emission scanning electron microscope (SEM). Prior to SEM imaging, the polymerized gel networks were lyophilized in a freeze dryer and the fractured surfaces were sputter‐coated with gold for 2 min at a current of 20 mA. The microstructures of networks near the fractured surfaces were imaged with the SEM with a tungsten source at 5 kV acceleration voltage.

### Mechanical Characterization of 3D Coll I Matrices

The fibrillar matrices of Coll I were reconstituted and probed in 1× PBS by an atomic force microscope (AFM, BioScope Catalyst, Bruker Corporation) coupled to a fluorescence microscope (TCS SP5 II, Leica). Prior to experiments, a polystyrene microsphere (15 µm diameter; Polysciences, Inc.) was glued to a cantilever (NP‐S type D nominal spring constant of 0.08 N m^−1^, Bruker Corporation) by a two‐component polyurethane glue (Bison International). The spring constant of the bead‐functionalized cantilever was calibrated per manufacturer's instructions. Matrix rigidities were quantified by repeatedly bringing the cantilever into contact with the surface of the specimens at an identical position (contact force ≤2 nN; approach‐retraction distance of 14 µm; approach velocity of 10 µm s^−1^). In each sample group, at least 16 force‐distance curves at three different positions were acquired from three independent experiments (Figure S12, Supporting Information).

### Transwell Migration/Invasion Assay

The classic transwell migration/invasion detection was also employed to analyze the ability of cancer cells in response to anti‐tumor drugs in a semiquantitative way based upon a protocol described in previous studies.^[^ ^29^ ^]^ Briefly, the breast cancer cell lines, that is, MDA‐MB‐231 and MCF‐7 cells, were first cultured in the 0.5% FBS‐contained medium for starvation for 12 h. Then, they were pretreated with different concentrations (0, 10, 20, and 30 nM) of PTX (Sigma‐Aldrich, USA) or the different concentrations (0 and 10 nM) of fulvestrant (Sigma‐Aldrich, USA) for another 2 h, washed with 1× phosphate‐buffered saline (PBS, Gibco, Life Technologies, USA), and harvested with trypsin (0.25%, Sigma‐Aldrich, USA). Next, aliquots (100 µL) of the cell suspension (5 × 10^5^ cells mL^−1^ in DMEM containing 1% (*v/v)* FBS and 1% (*v/v*) 100× penicillin/streptomycin solution) were pipetted into Costar Transwell insert (Corning, New York, USA) precoated with Matrigel (1:8 diluted with DMEM, Corning, New York, USA) and placed into each well in a 24‐well cell culture plate supplemented with 600 µL of DMEM containing 20% (*v/v*) FBS and 1% (*v/v*) 100× penicillin/streptomycin solution. The cells were allowed to migrate for 24 h, then fixed with 4% formaldehyde (Sigma‐Aldrich, USA) for 30 min, stained with 0.1% crystal violet (Sigma‐Aldrich, USA) solution in 1× PBS for 30 min and finally washed with deionized water. The membranes were excised from the transwell inserts, mounted in the Vectashield mounting medium (Vector Laboratories, Inc., Burlingame, USA) and imaged with an inverted fluorescence microscope (CKX41, Olympus, Japan). To quantify the cell number, five images were taken from each sample originating from three independent experiments.

### Methyl Thiazolyl Tetrazolium‐Based Cell Viability Assay

Aliquots (100 µL) of cell suspension (1 × 10^5^ cells mL^−1^ in DMEM containing 10% (*v/v*) FBS, 1% (*v/v*) 100× penicillin/streptomycin solution) were pipetted into a 96‐well microplate (Corning, New York, USA). The cells were first treated with 0.5% FBS‐contained medium for starvation for 12 h, stimulated with different concentrations (0, 10, 20, and 30 nM) of PTX (Sigma‐Aldrich, USA) for another 2 h, and washed with 1× PBS (Gibco, Life Technologies, USA). Subsequently, 10 µL Methyl Thiazolyl Tetrazolium (MTT) (5 mg mL^−1^, Sigma‐Aldrich, USA) was added into each well and the cells were further cultured at 37 °C, 5% CO_2_ and 95% humidity for 4 h, following by the addition of 100 µL formazan dissolving solution. After the formazan crystals were completely dissolved, the absorbance at 570 nm was recorded using a microplate reader. For the sake of statistical analysis, at least three independent MTT tests were performed in the current experiments.

### Quantitative Analysis of Cell Morphology

Cell morphology was analyzed to explore the effects of anti‐tumor drugs, for example, PTX, on cellular functions and behaviors. To this end, the cells were first treated with PTX (0, 10, 20, and 30 nM) for 2 h, washed with 1× PBS and fixed in 4% (*w/v*) paraformaldehyde (Sigma‐Aldrich, USA) for 20 min. Then, they were permeabilized with 0.1% (*v/v*) Triton X100 (Sigma‐Aldrich, USA) and blocked in 2% (*w/v*) BSA (Sigma‐Aldrich, USA) at room temperature for 2 h. Next, the cells were stained with Alexa Fluor 555 Phalloidin (dilution 1:200 in 1× PBS; Invitrogen, USA), and anti‐*α*‐Tubulin‐Alexa Fluor 488 (dilution 1:100 in 2% (*w/v*) BSA; Invitrogen, USA) at room temperature for 3 h, and DAPI (4′, 6‐diamidino‐2‐phenylindole, dilution 1:10 000 in 1× PBS; Invitrogen, USA) at room temperature for 30 min. The immunostained cells were imaged by an inverted confocal laser scanning microscope equipped with 20× /NA 0.75 objective (Nikon A1, Japan). Accordingly, the spreading areas and aspect ratios of the cells were determined via the ImageJ software (NIH, USA). For statistical analysis, at least four images were investigated from three independent experiments.

### Western Blot Assay

Western blotting was employed to detect the expression levels of ER‐*α* and vinculin in MCF‐7 cells treated with fulvestrant (0 and 10 nM). In brief, the cells were first rinsed with pre‐chilled 1× PBS buffer (pH 7.4) to remove soluble factors, then lysed with radio immunoprecipitation assay lysis (RIPA) buffer containing 0.5 M Tris‐HCl (pH 7.4), 1.5 M NaCl, 2.5% deoxycholic acid, 10% NP‐40, 10 mM EDTA, and protease inhibitor mixture. The total protein mixture was separated through SDS‐PAGE and transferred to polyvinylidene difluoride membranes using a Bio‐Rad semi‐dry electrophoretic cell. After the membranes were incubated with anti‐ER*α* antibody (dilution 1:750 in 1× PBS; Abcam, USA) and anti‐vinculin antibody (dilution 1:400 in 1× PBS; Abcam, USA), the target antibodies were labeled with a horseradish peroxidase (HRP)‐conjugated IgG antibody. Finally, the enhanced chemiluminescence (Pierce) was utilized for immunoreactive protein visualization.

### Immunofluorescence Analysis

The effect of anti‐tumor drugs on the expression of ER*α* and vinculin in MCF‐7 cells was analyzed after 2 h pretreatment with fulvestrant (0 and 10 nM). First, those treated cells were washed with 1× PBS, fixed in 4% (*w/v*) paraformaldehyde (Sigma‐Aldrich, USA) for 20 min, permeabilized with 0.1% (*v/v*) Triton X100 (Sigma‐Aldrich, USA) and blocked in 2% (*w/v*) BSA (Sigma‐Aldrich, USA) at room temperature for 2 h. Afterward, they were incubated with anti‐ER*α* antibody (dilution 1:150 in 1× PBS, Abcam, USA) or recombinant anti‐vinculin antibody (dilution 1:150 in 1× PBS, Abcam, USA) overnight at 4 °C, and subsequently incubated with secondary FITC‐conjugated antibodies for 2 h, respectively. Next, their cytoskeletons were stained with Alexa Fluor 555 Phalloidin (dilution 1:200 in 1× PBS, Invitrogen, USA) at room temperature for 3 h, whereas the nuclei were counterstained with DAPI (dilution 1:10 000 in 1× PBS, Invitrogen, USA) at room temperature for 30 min. After each fixation, permeabilization and staining steps, the treated cells were rinsed 4 times with 1× PBS in the experiments. The immunofluorescent cells were visualized using an inverted confocal laser scanning microscope equipped with 20× /NA 0.75 objective (Nikon A1, Japan), which confirmed that the expression of vinculin was decreased after downregulation of nuclear expression of ER*α* in the MCF‐7 cells.

### Quantitative Analysis of Electric Fields Created by Interdigitated Electrodes

Essentially, the electric fields induced by the interdigitated electrodes satisfied the classical Laplace's equation ∇^2^ *φ* = 0 with *φ* denoting the electric potential. This differential equation was numerically solved via the well‐established partial differential equation toolbox in Matlab (MathWorks, USA). Figure 1B presented spatial distributions of the electrical potentials and electric fields around the interdigitated electrodes.

### Quantitative Characterization of Pore Size in 3D Collagen Matrices

The network topology of 3D collagen matrices was characterized by quantifying a series of fluorescence images of the 3D matrices using a well‐developed image processing algorithm.^[^ ^54^ ^]^ In brief, 3D collagen matrices with different collagen concentrations (2.5, 3.0, 3.5, and 4.0 mg mL^−1^) were first prepared in experiment. For subsequent analyses of network topology, the collagen network structures were labeled with 5(6)‐TAMRA‐SE (5‐(and‐6)‐carboxytetramethylrhodamine succinimidyl ester, Invitrogen, Carlsbad, USA) per manufacturer's instructions. Then, the 3D fluorescently labeled collagen networks were imaged with an inverted confocal laser scanning microscope (Nikon A1, Japan) equipped with 20× /NA 0.75 objective (Nikon A1, Japan), as shown in Figure 2C.

As described in the literature,^[^ ^54^ ^]^ the well‐established image processing algorithm consisted of several consecutive steps to extract the key information on network topology of the collagen matrices, that is, pore size, fibril diameter and fibril content. The first step was to transform the 2D fluorescence images into a series of binary images, as exhibited in Figure S5A, Supporting Information, and perform image segmentation into pore and fibril segments with the aid of Matlab (MathWorks, USA). Subsequently, an erosion algorithm was employed to determine the pore areas, which filled up all the segmented pores with circular disks whose diameters were beforehand set as 1, 5, 10, and 15 pixels, respectively, as displayed in Figure S5B, Supporting Information. The corresponding eroded pore areas were found to be 93.39%, 67.45%, 36.84%, and 20.51%. The mean pore diameter was estimated as the specific disk diameter corresponding to 50% of the eroded pore area.

### Quantification of Cell Migration/Invasion in 3D Collagen Matrices Based on Confocal Microscopy

The ability of cell migration/invasion in 3D collagen matrices in response to chemoattractant gradients were quantitatively detected by an inverted confocal laser scanning microscope (Nikon A1, Japan). To facilitate subsequent image analysis, the cells were first labeled as green with Cell Tracker Green CMFDA (5‐chloromethylfluorescein diacetate, ThermoFisher Scientific, USA) per manufacturer's instructions. The spatio‐temporal dynamics of the collective cell migration/invasion in the 3D matrices encapsulated into the chip chambers were monitored through 3D volumetric imaging based on the inverted confocal laser scanning microscope with 20× /NA 0.75 objective. The time interval of 3D imaging was 30 min whereas the sizes of these acquired time‐series volumetric images along X, Y and Z directions were 1000, 1000, and 31 voxels, respectively, as shown in Videos S2 and S3, Supporting Information. The total time of 3D imaging was 180 min in each experiment. Subsequently, a self‐written Matlab (MathWorks, USA) codes were untitled to statistically determine the spatial coordinates of the migrating and invading cells labeled with Cell Tracker Green CMFDA at different time points. In this way, this work might quantify the time‐related displacements and migration/invasion speeds of the cells away from the bottom of the chip chambers, as shown in Figure 4.

### Hematoxylin and Eosin and Alpha Fetoprotein Staining

H&E staining was performed to visualize the distribution of cells and morphological changes in the hepatic carcinoma tissues and para‐carcinoma tissues. In experiments, they were fixed by 4% paraformaldehyde for 48 h. Subsequently, they were dehydrated with a graded series of ethanol (50%, 70%, 80%, 95%, and 100%) and embedded in paraffin. The paraffin‐embedded tissues were sectioned into 5 µm thick slices and tiled on glass slides. Then, those sections were deparaffinized with dimethyl benzene and rehydrated with 100%, 95%, 80%, and 70% ethanol. On the other hand, immunohistochemical (IHC) staining was carried out using a polyclonal antibody against AFP (Proteintech, 14550‐1‐ap). The paraffin‐embedded tissue sections were deparaffinized and rehydrated. Two percent H_2_O_2_‐methanol was employed to ablate the endogenous peroxidase, and 5% normal horse serum was utilized to suppress nonspecific protein binding. The tissue sections were incubated at 37 °C for 1 h and then incubated with primary antibodies at 4 °C overnight. Afterward, they were washed and incubated with secondary biotinylated goat anti‐rabbit IgG at 37 °C for 1 h, and incubated with avidin‐biotin complex at 37 °C for 45 min. Color was developed with diaminobenzidine tetrahydrochloride.

### Collection of Human Hepatic Carcinoma Tissue Samples

A piece of de‐identified fresh human hepatic carcinoma tissue was acquired from Fifth Central Hospital of Tianjin, Tianjin, China. The entire study protocols were reviewed and approved by the Ethics Committee of Fifth Central Hospital of Tianjin (Tianjin, 300 450, China, Approval Number: TJWZXLL2022039). Written informed consent was obtained from the liver cancer patient according to CARE guidelines and in compliance with the Declaration of Helsinki principles.

### Statistical Analysis

All results were represented as Mean ± SD from at least three independent samples. Significant differences between data sets were calculated using an unpaired *t*‐test at a *p*‐level of 5%.

## Conflict of Interest

The authors declare no conflict of interest.

## Author Contributions

J.H. and N.J. conceived the idea and designed the study. N.J., Y.M.H., and J.H. developed the chip systems, conducted the experiments and performed the image analysis. L.X., S.W., X.D., and J.H. developed the theoretical models, wrote Matlab program codes and analyzed the experimental data. N.J. and Y.X.H. fabricated the high throughput chips. N.J., Y.M.H., J.D., X.L., and Z.L. designed and conducted the in vitro and ex vivo experiments and analysis. All these authors discussed and interpreted the experimental and theoretical results, and participated in writing and editing the manuscript.

## Supporting information

Supporting Information

Supplemental Video 1

Supplemental Video 2

Supplemental Video 3

## Data Availability

The data that support the findings of this study are available from the corresponding author upon reasonable request.
