# Supplementary material for: High‐Throughput Electromechanical Coupling Chip Systems for Real‐Time 3D Invasion/Migration Assay of Cells
Source: Adv Sci (Weinh). 2023 Apr 23;10(19):2300882. doi: 10.1002/advs.202300882 (PMC10323643; doi:10.1002/advs.202300882)
Supplement: Supplementary file 1 — Supporting Information [file ADVS-10-2300882-s002.pdf]

ADVANCED SCIENCE
Open Access

Supporting Information

for *Adv. Sci.*, DOI 10.1002/advs.202300882

High-Throughput Electromechanical Coupling Chip Systems for Real-Time 3D Invasion/Migration Assay of Cells

*Nan Jiang, Liang Xu, Yiming Han, Shuyi Wang, Xiaocen Duan, Jingyao Dai, Yunxing Hu, Xiaozhi Liu, Zhiqiang Liu and Jianyong Huang**

Supporting Information

High-throughput electromechanical coupling chip systems for real-time 3D invasion/migration assay of cells

*Nan Jiang, Liang Xu, Yiming Han, Shuyi Wang, Xiaocen Duan, Jingyao Dai, Yunxing Hu, Xiaozhi Liu, Zhiqiang Liu, and Jianyong Huang**

This PDF file includes:

Figures S1 to S22
Tables S1 to S5
Legends for videos S1 to S3

Supplementary Figures

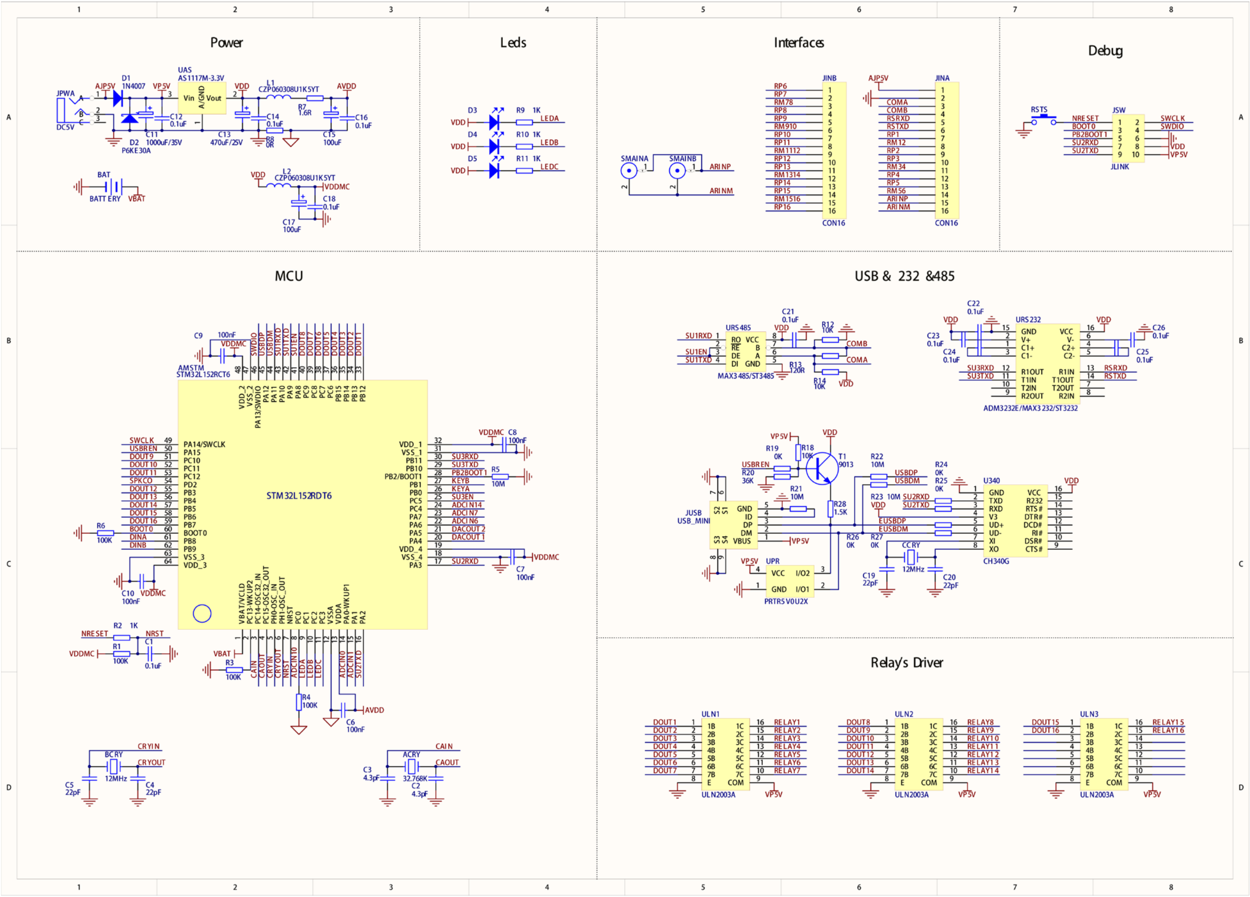

Figure S1. The circuit diagram of the electromechanical coupling chip systems.

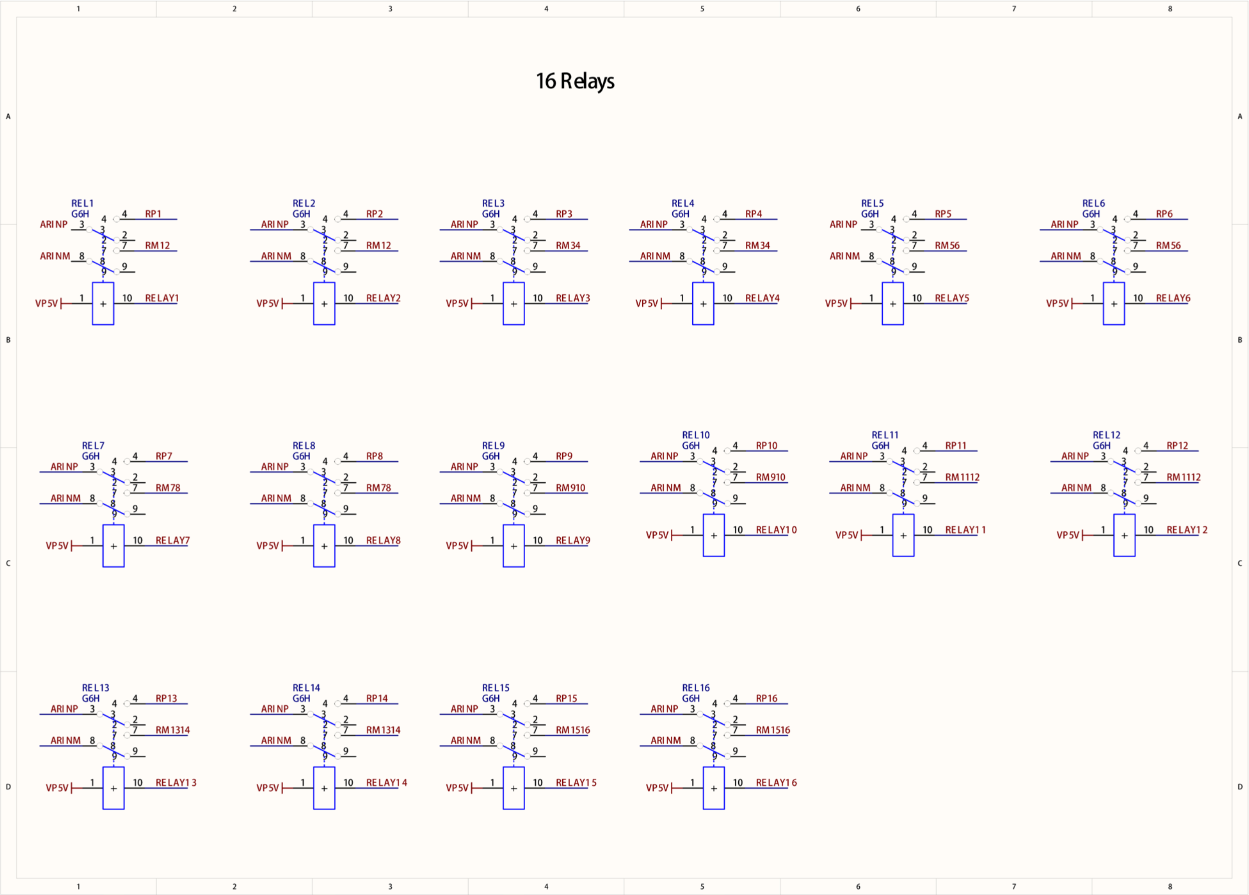

Figure S2. The circuit diagram of the designed relays in the electromechanical coupling chip systems.

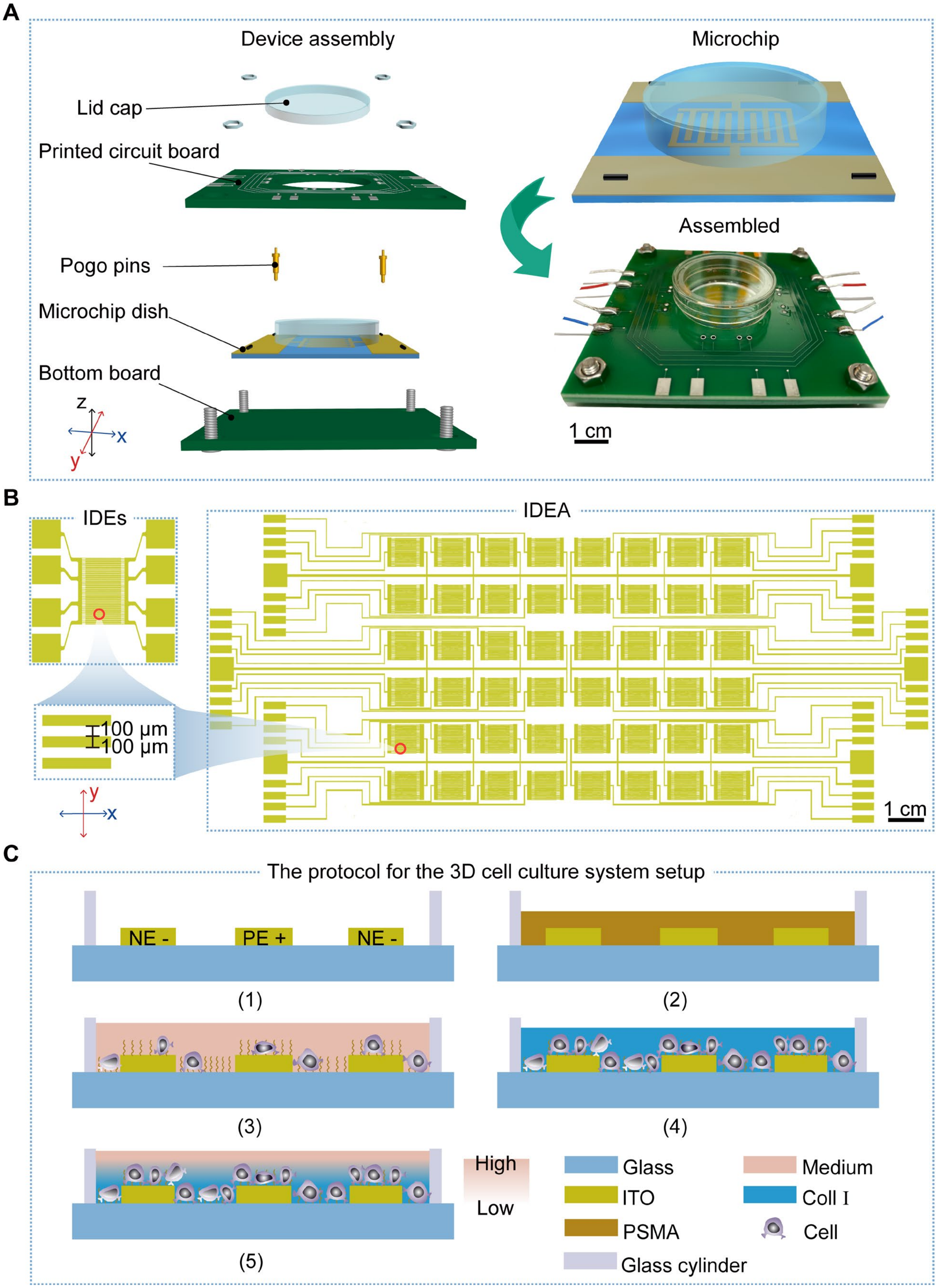
A
Device assembly
Microchip
Lid cap
Printed circuit board
Pogo pins
Microchip dish
Bottom board
Assembled
1 cm
z
x
y
B
IDEs
IDEA
100 μm
100 μm
1 cm
y
x
C
The protocol for the 3D cell culture system setup
NE -
PE +
NE -
(1)
(2)
(3)
(4)
(5)
High
Low
Glass
ITO
PSMA
Glass cylinder
Medium
Coll I
Cell

Figure S3. Fabrication of electromechanical coupling chips.

(A) Schematic and photographs of the assembly process of a single-channel biochip. A designed PCB was connected to the fabricated ITO electrodes through the pogo pins in this chip, and then connected to an electrical impedance spectrometer for subsequent experiments.

(B) Design of high-throughput IDEs on an ITO glass substrate, which contained 48 independent IDEA channels.

(C) Schematic diagram of 3D cell culture on the electromechanical coupling chip chamber. (Step 1 and 2) ITO glass slides with designed IDEA were surface-functionalized with PSMA so that the collagen matrices can be covalently immobilized on it; (Step 3) Cell culture on the ITO substrate with IDEA; (Step 4) In situ formation of 3D collagen matrix; (Step 5) Addition of chemokines to create a chemoattractant gradient in the 3D culture environment.

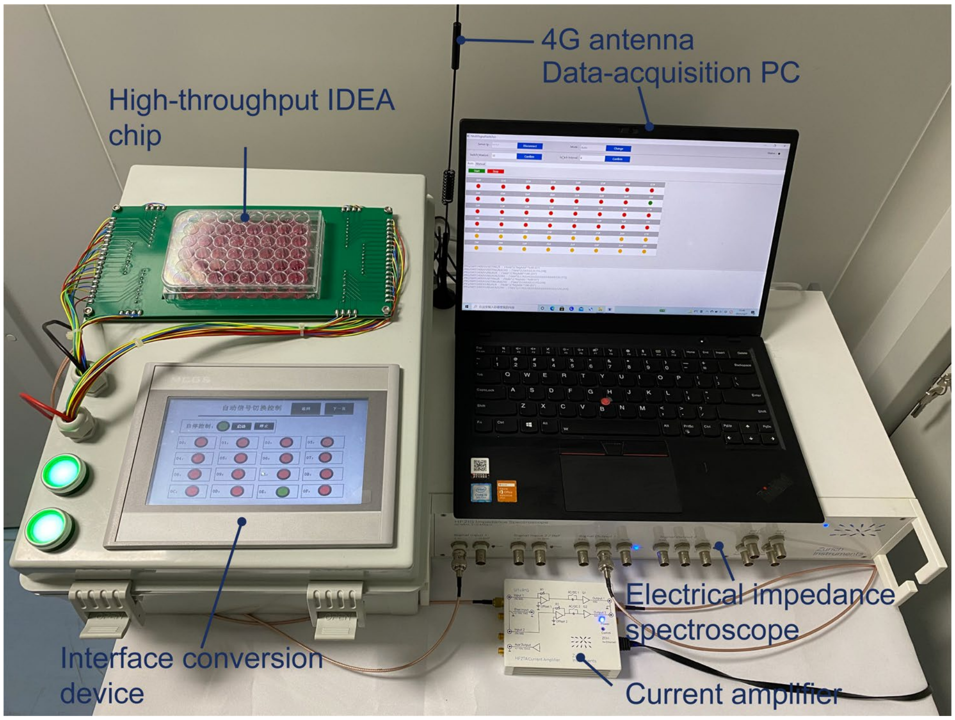

Figure S4. Experimental system setup. Developed experimental setup, which included high-throughput IDEA chips, interface conversion device, electrical impedance spectroscope, data acquisition system, etc. In actual experiments, the IDEA chips were placed in a standard cell culture incubator containing 5% CO2 and 95% humidity at 37 °C.

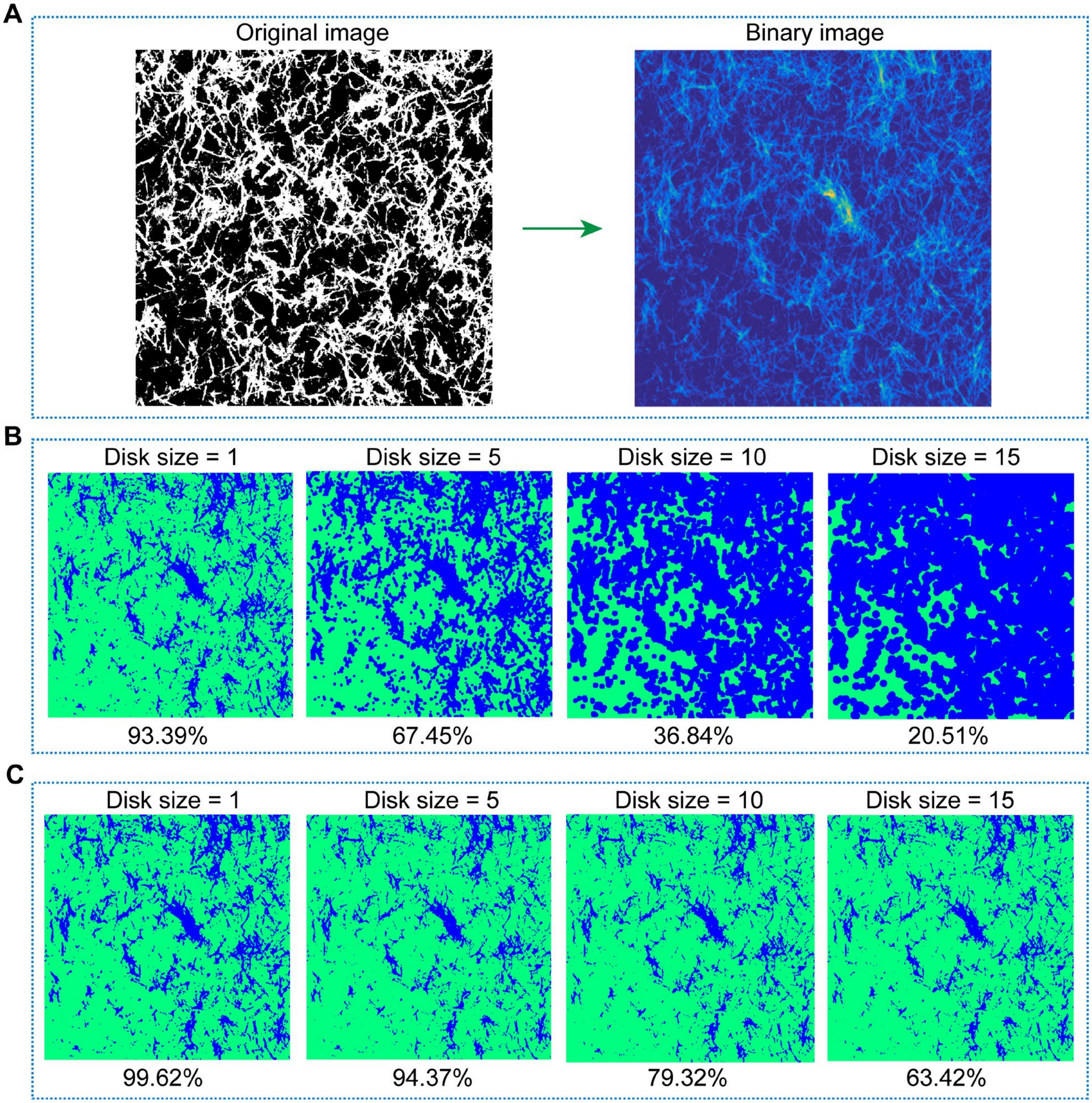

Figure S5. Determination of pore areas in the collagen matrices based on an erosion algorithm.

(A) Transformation of original images into binary images to numerically evaluate network topology of the collagen matrices.

(B) Erosion steps for determination of the pore sizes. The erosion algorithm randomly filled all segmented pores with disks of a predefined size. By increasing the disk size, the pore area decreased due to spatial confinement of the pores. At each erosion step, the total area of the existing pores was determined accordingly.

(C) Dilation steps for determination of the pore sizes.

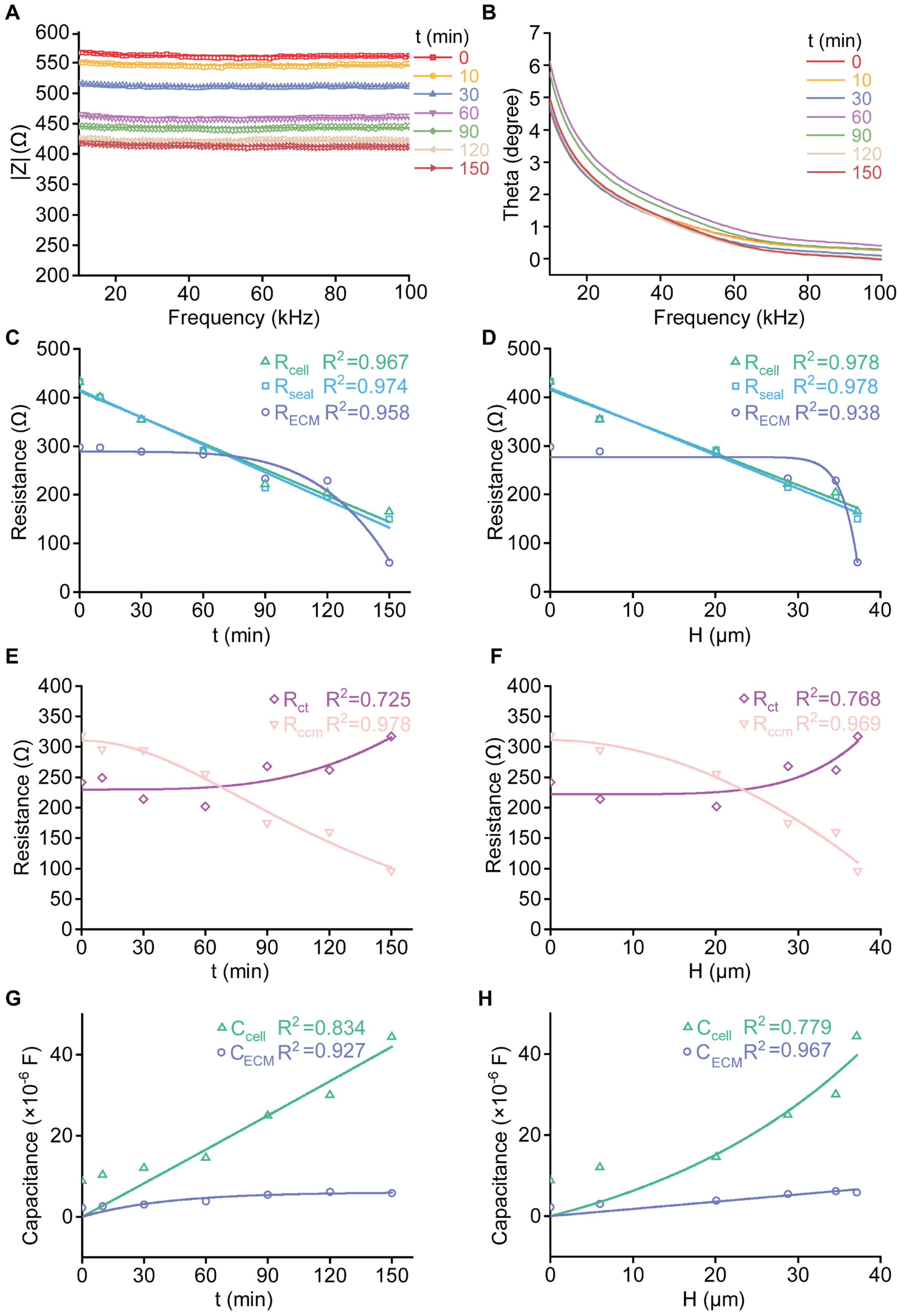
A
B
C
D
E
F
G
H
|Z| (Ω)
Frequency (kHz)
t (min)
0
10
30
60
90
120
150
Theta (degree)
Resistance (Ω)
R_cell R²=0.967
R_seal R²=0.974
R_ECM R²=0.958
R_cell R²=0.978
R_seal R²=0.978
R_ECM R²=0.938
H (μm)
R_ct R²=0.725
R_ccm R²=0.978
R_ct R²=0.768
R_ccm R²=0.969
Capacitance (×10⁻⁶ F)
C_cell R²=0.834
C_ECM R²=0.927
C_cell R²=0.779
C_ECM R²=0.967

Figure S6. Spatiotemporal changes in complex impedance signals in response to 3D cell migration and invasion in the chip systems.

(A) The magnitudes of representative complex impedance spectra in the range of 10~100 kHz measured at the specific time points of 0, 10, 30, 60, 90, 120 and 150 min after 3D migration/invasion of MDA-MB-231 cells cultured in 4.0 mg/ml Coll I matrix, respectively. The discrete data points represented the actually measured results while the solid curves denoted the corresponding fitting results based on the proposed equivalent circuit model.

(B) The corresponding phase characteristics of the complex impedance spectra.

(C, D) The changes in R_{cell}, R_{seal} and R_{ECM} with time and average distance these cells moved away from the underlying IDEs, respectively.

(E, F) The changes in R_{ct} and R_{ccm} with time and average distance these cells moved away from the underlying IDEs, respectively.

(G, H) The changes in C_{cell} and C_{ECM} with time and average distance these cells moved away from the underlying IDEs, respectively. The discrete data were obtained by fitting the measured complex impedance spectra with the proposed equivalent circuit model, whereas the solid curves were the corresponding data fitting results.

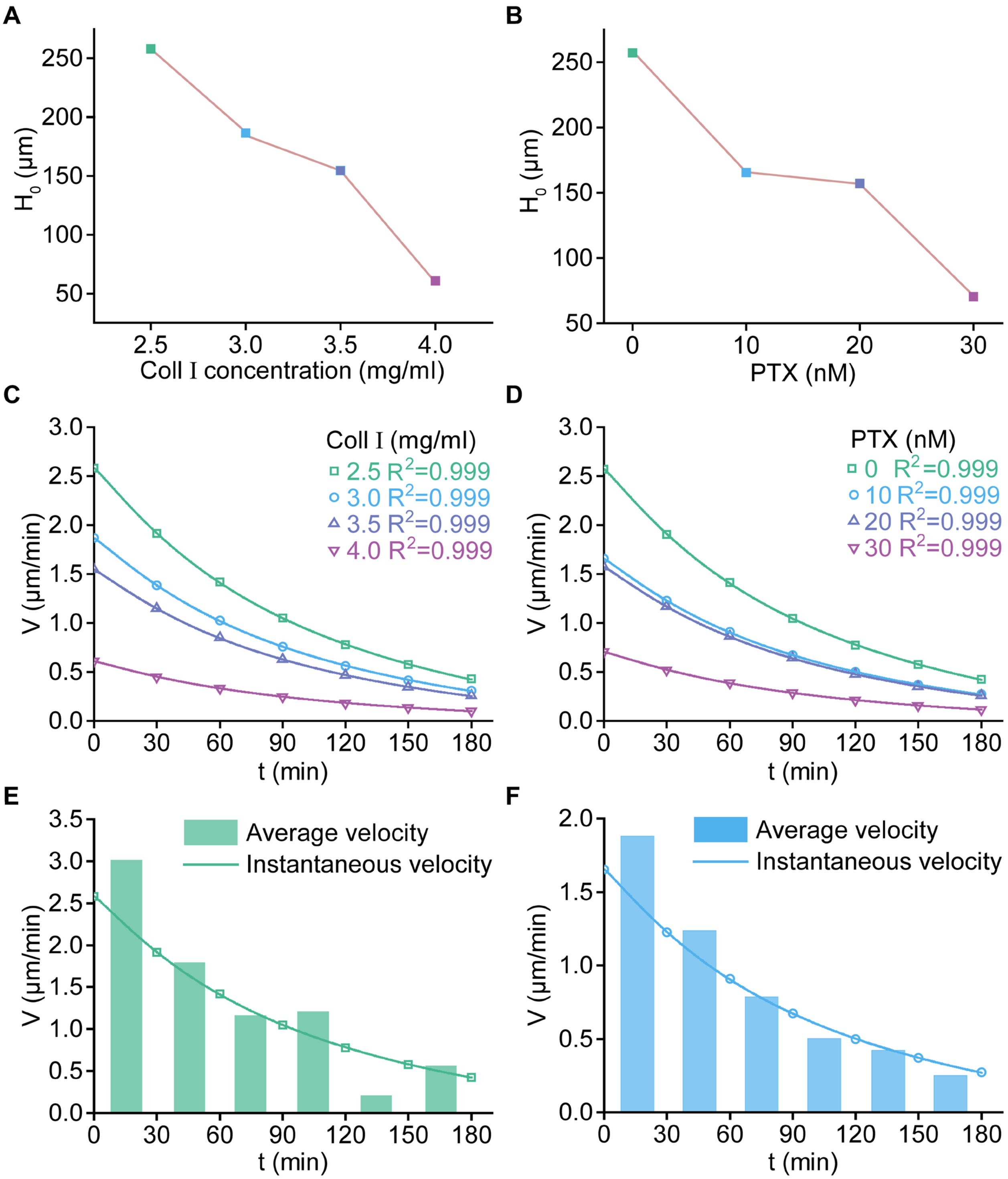

Figure S7. Spatiotemporal dynamic changes in 3D cell migration/invasion in response to the concentrations of Coll I and PTX in the chip systems.

(A, B) Steady-state invasion/migration distances away from IDEs of the 3D invading/migrating cells in the collagen matrices whose monomer concentrations were 2.5, 3.0, 3.5 and 4.0 mg/ml

(A), or encapsulated in 2.5 mg/ml collagen matrix, which were pretreated with different concentrations (10, 20 and 30 nM) of PTX (B), respectively.

(C, D) Instantaneous invasion/migration velocities of the MDA-MB-231 cells grown in 3D collagen matrices in response to the specific chemoattractant gradient (C), or encapsulated in 2.5 mg/ml collagen matrix, which were pretreated with different concentrations (10, 20 and 30 nM) of PTX (D), respectively.

(E, F), Quantitative comparisons between the average velocities per half hour and the instantaneous velocities of the 3D invading/migrating cells encapsulated in 2.5 mg/ml collagen matrix, which were pretreated with 0 (E) and 10 nM PTX (F), respectively.

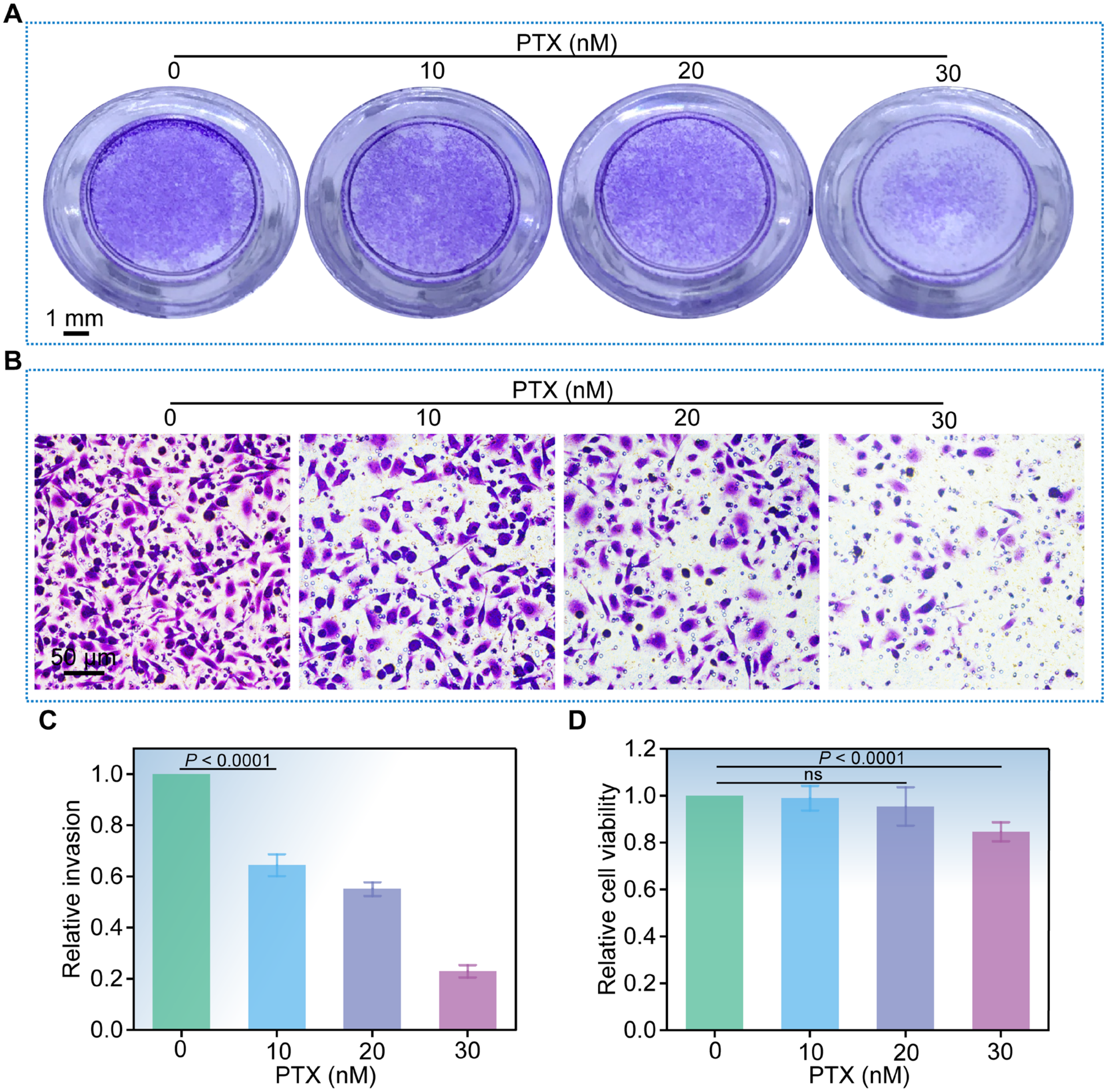

Figure S8. A transwell assay showed relative invasion ability of MDA-MB-231 cells pretreated with the different concentrations (0, 10, 20 and 30 nM) of PTX.

(A-C) The relative invasion analysis was performed at least in triplicate with 5 positions per sample.

(D) A methyl thiazolyl tetrazolium (MTT)-based cell viability assay displayed relative cell viability of MDA-MB-231 cells, which were also pretreated with the same concentration of PTX. Data are shown from at least 3 independent experiments ($n \geq 3$) with Mean ± SD. ns - no significant difference.

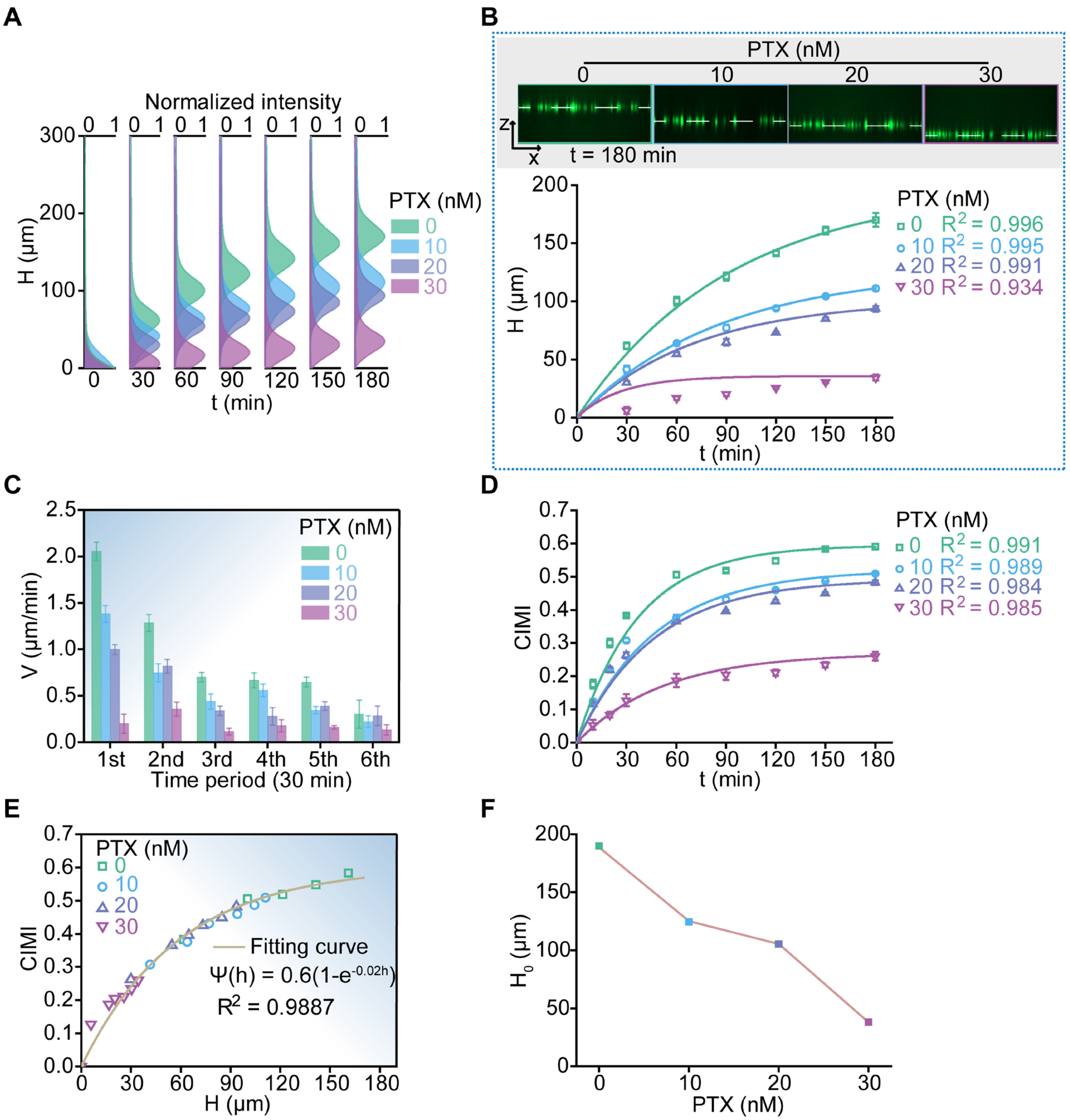

Figure S9. Efficacy evaluation of anti-tumor drugs based on 3D-CIMA. Prior to the experiments, MDA-MB-231 cells encapsulated in 3.0 mg/ml collagen matrix were pretreated with different concentrations (10, 20 and 30 nM) of paclitaxel (PTX, Sigma-Aldrich, USA), respectively.

(A) Spatiotemporal distributions of 3D invading/migrating cells that were beforehand labelled as green with Cell Tracker Green CMFDA. The Gaussian-like spatial distributions exhibited 3D collective cell invading/migrating behavior at different time points.

(B) Inhibition of PTX on invasion/migration of the MDA-MB-231 cells in the collagen matrix, which was quantified with the conventional confocal fluorescence imaging method. The four insets on the upper part showed spatial positions of the green fluorescence-labelled cells after 180 min of upward invasion/migration in the collagen matrix, where the PTX concentrations were 0, 10, 20 and 30 nM, respectively.

(C) Average invasion/migration velocities per half hour of the cells treated with PTX of 0, 10, 20 and 30 nM, respectively.

(D) Time-dependent CIMI data for the cells treated with PTX of 0, 10, 20 and 30 nM, respectively. Data were shown as Mean ± SD (n = 3).

(E) Quantitative comparison between the invasion/migration distances away from IDEs and the corresponding CIMIs quantified by 3D-CIMA.

(F) Steady-state invasion/migration distances away from IDEs of the 3D invading/migrating cells encapsulated in 3.0 mg/ml collagen matrix were pretreated with different concentrations (10, 20 and 30 nM) of PTX, respectively.

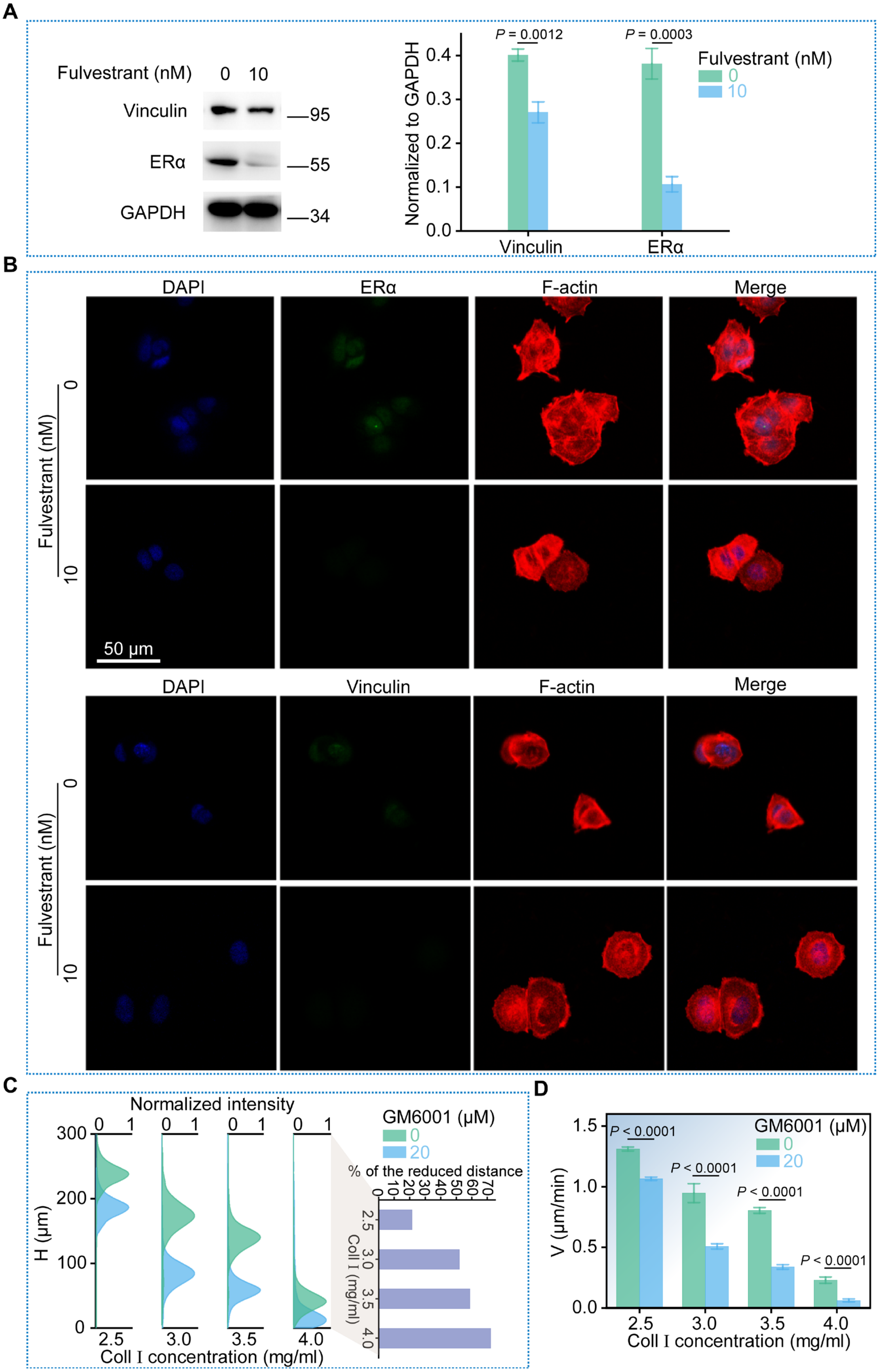
A
Fulvestrant (nM) 0 10
Vinculin
ERα
GAPDH
95
55
34
Normalized to GAPDH
P = 0.0012
P = 0.0003
Fulvestrant (nM)
0
10
Vinculin
ERα
B
DAPI
ERα
F-actin
Merge
Fulvestrant (nM)
0
10
50 μm
DAPI
Vinculin
F-actin
Merge
C
Normalized intensity
H (μm)
GM6001 (μM)
0
20
% of the reduced distance
Coll I (mg/ml)
Coll I concentration (mg/ml)
D
V (μm/min)
P < 0.0001
GM6001 (μM)
0
20
Coll I concentration (mg/ml)

Figure S10. Antineoplastic drugs orchestrated invasion/migration of breast cancer cells in 3D collagen matrix.

(A) Western blotting was conducted to detect the effect of fulvestrant on the protein levels of vinculin and ERα in MCF-7 cells. The results were normalized to GAPDH (n = 3).

(B) Confocal assay was performed to detect the effect of fulvestrant on the expression of ERα and vinculin in the MCF-7 cells. The cell nuclei, actin filaments, ERα and vinculin were stained blue, red and green, respectively.

(C) Spatiotemporal distribution of 3D invading/migrating cells that were beforehand labelled as green with Cell Tracker Green CMFDA after 180 min of upward invasion/migration in the collagen matrix, which displayed a dose-dependent upward migration delay. The Gaussian-like spatial distributions exhibited 3D collective invasion/migration process of the MDA-MB-231 cells after treatment with 20 μM GM6001 in different collagen matrices.

(D) Average invasion/migration velocities after 180 min of upward invasion/migration of the MDA-MB-231 cells treated with GM6001 of 0 and 20 μM, respectively.

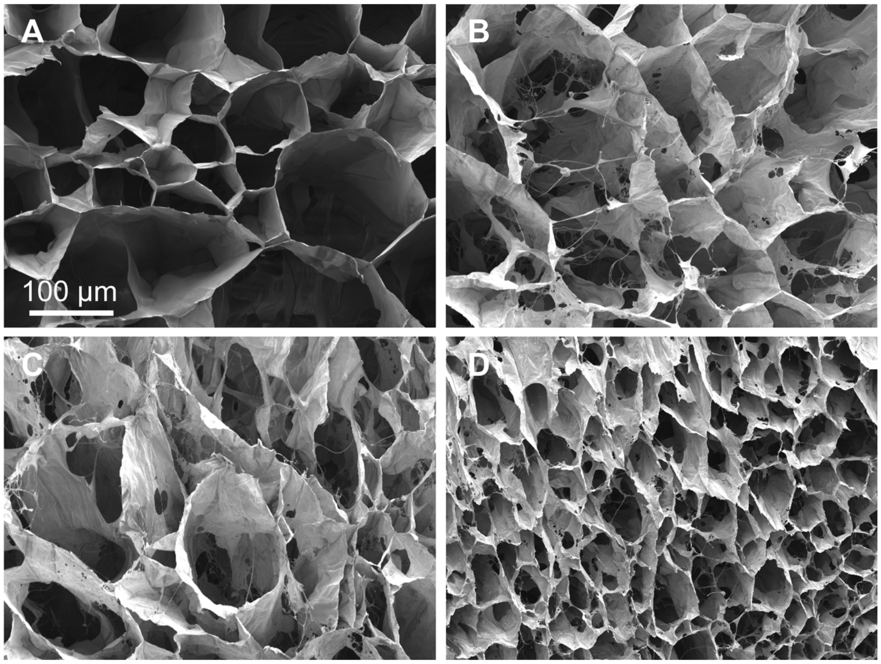

Figure S11. Typical SEM images of Coll I matrices, which were reconstituted at the concentrations of (A) 2.5 mg/ml, (B) 3.0 mg/ml, (C) 3.5 mg/ml and (D) 4.0 mg/ml, respectively.

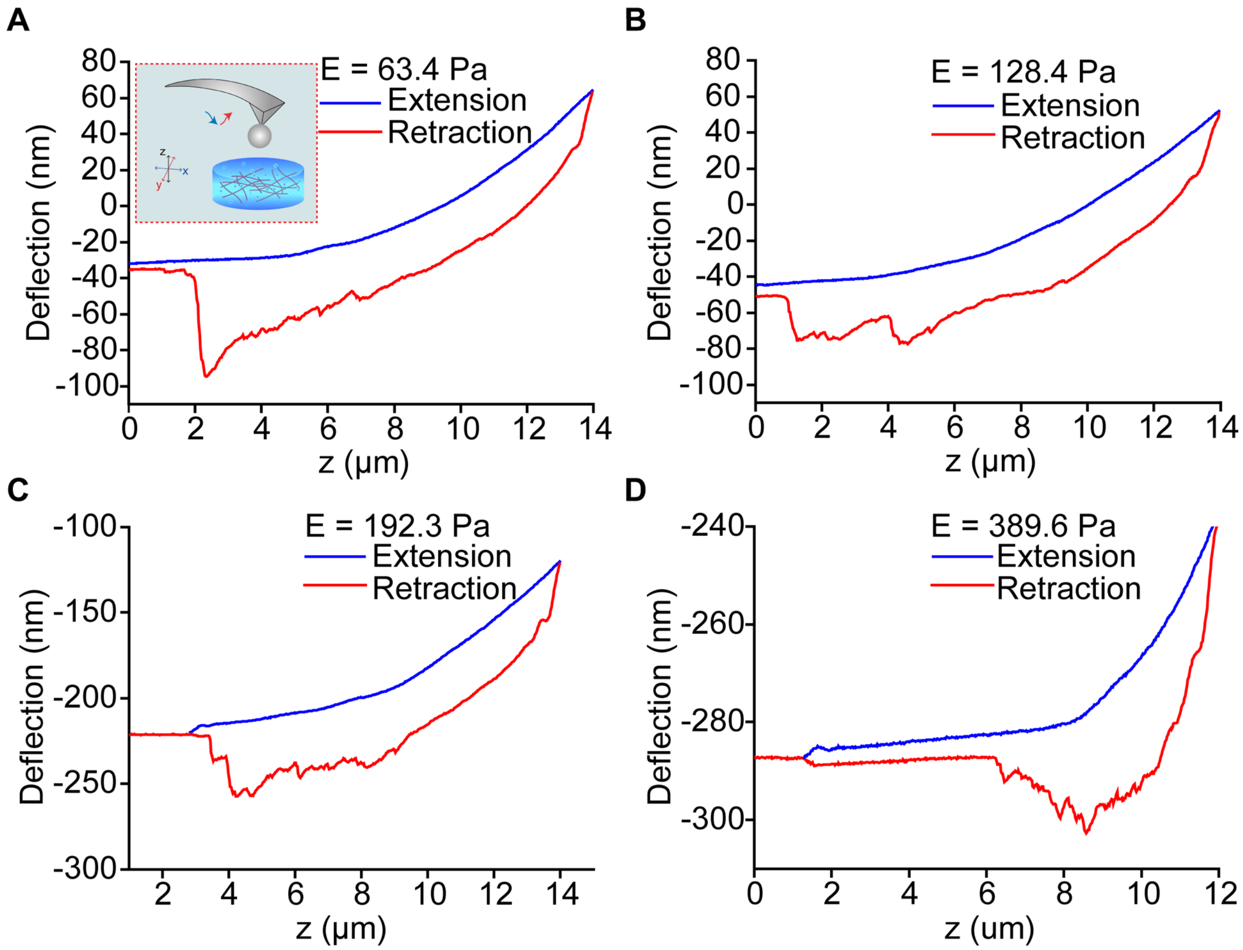

Figure S12. Representative plots of force-indentation curves for mechanical characterization of Coll I matrices by AFM. Coll I matrices were reconstituted at concentrations of (A) 2.5 mg/ml, (B) 3.0 mg/ml, (C) 3.5 mg/ml and (D) 4.0 mg/ml, respectively.

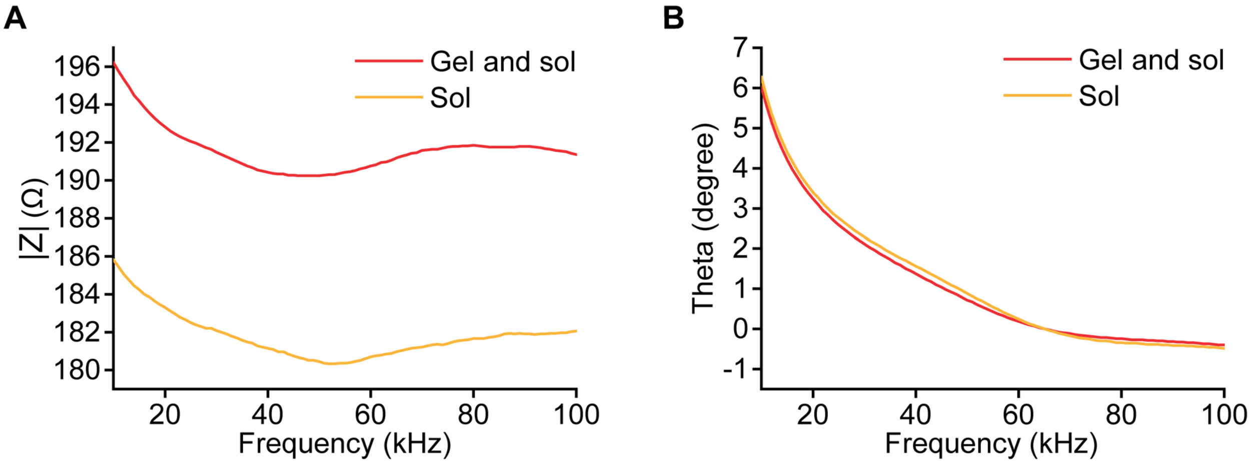

Figure S13. Representative plots of complex impedance spectroscopy (A) and phase characteristic (B) for only complete culture medium or 2.5 mg/ml Coll I matrix in complete culture medium, respectively.

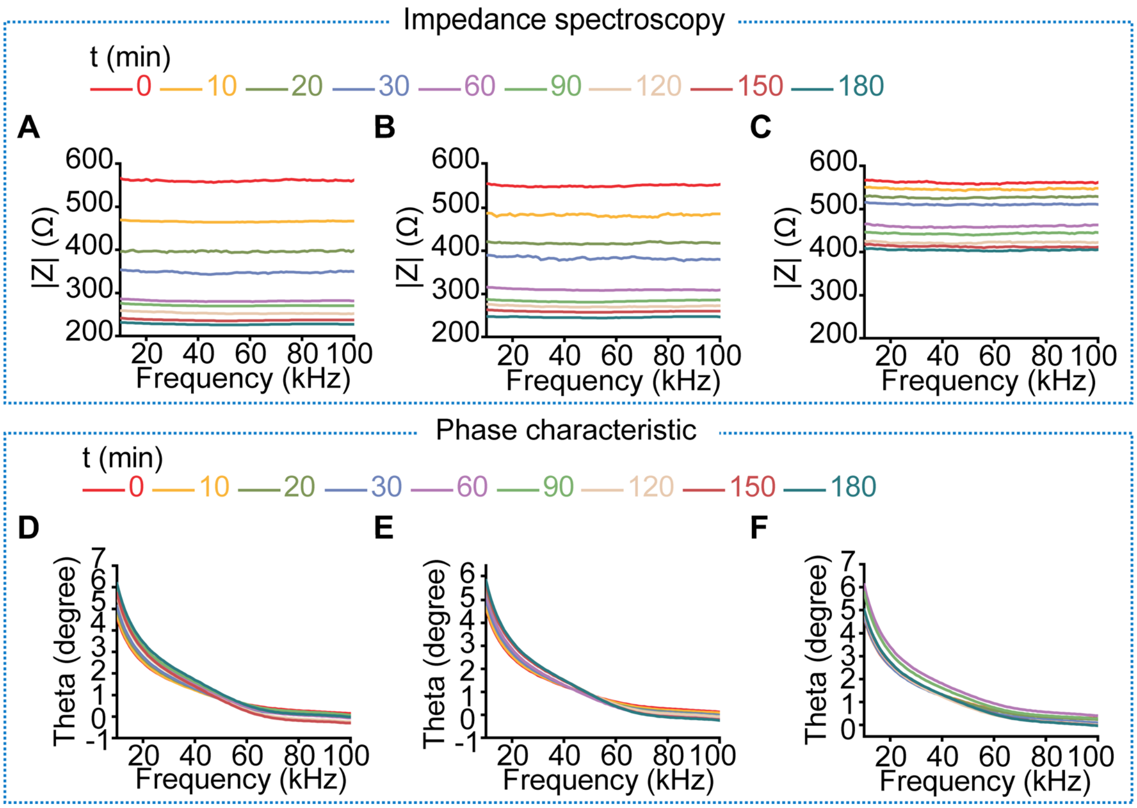

Figure S14. Results of 3D-CIMA for monitoring collective cell migration in 3D Coll I matrices of different monomer concentrations ((A, D) 3.0 mg/ml, (B, E) 3.5 mg/ml and (C, F) 4.0 mg/ml). 3D-CIMA data are presented as impedance (Ω) and phase (degree).

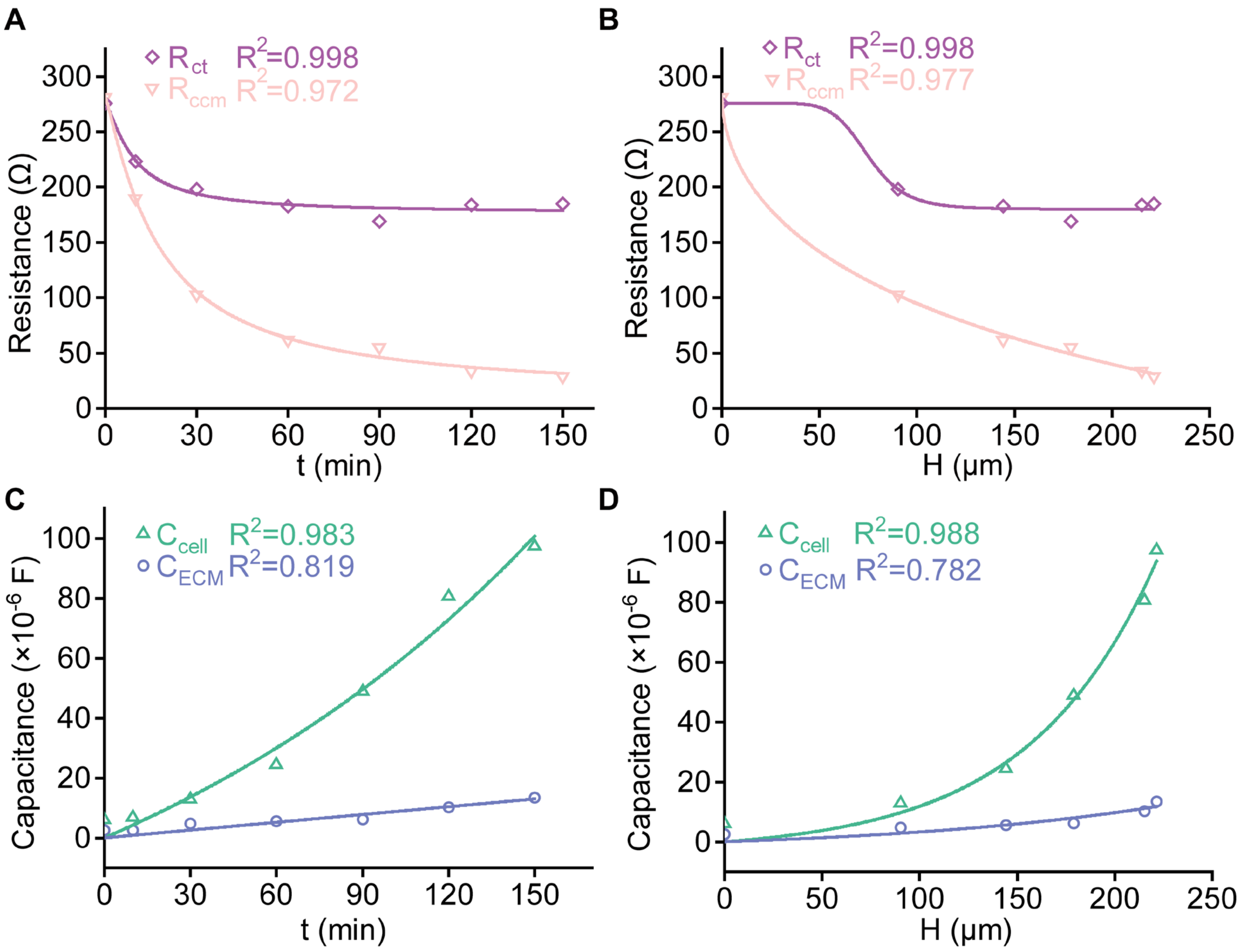

Figure S15. Cell invasion/migration-induced changes in resistance and capacitance of the electrical elements in the developed 3D-CIMA.

(A, B) Spatiotemporal dynamics of the resistance of cell culture media (i.e., R_{ccm}) and the charge transfer resistance (i.e., R_{ct}), where the cells underwent an upward collective cell invasion/migration in 2.5 mg/ml Coll I matrix from the top surface of IDEA.

(C, D) Spatiotemporal dynamics of the membrane capacitance (i.e., C_{cell}) and the capacitance of ECM (i.e., C_{ECM}). The discrete data denoted the results numerically solved through the equivalent circuit model whereas the solid curves were the fitting results.

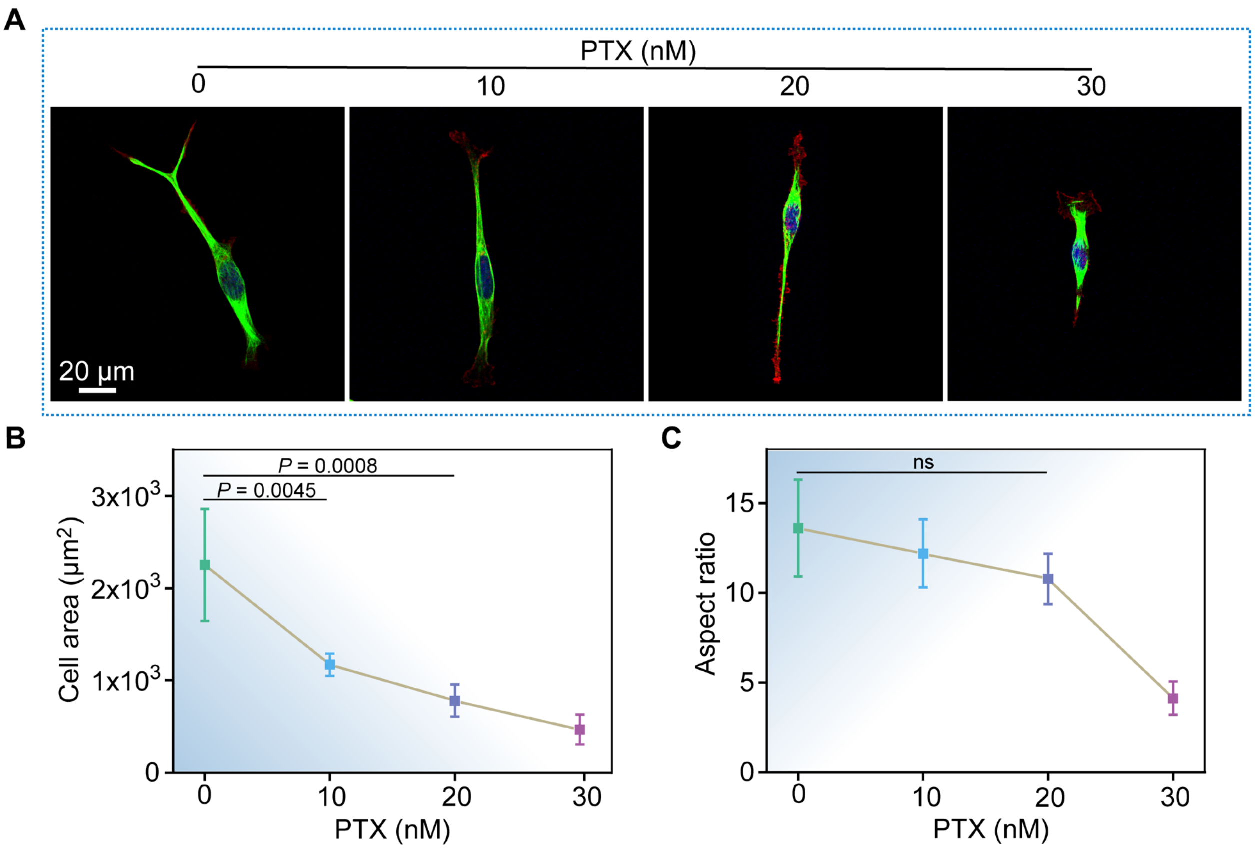

Figure S16. Effect of PTX on morphology and invasiveness of MDA-MB-231 cells.

(A) Representative images of single MDA-MB-231 cell after 2 h pretreatment with PTX (0, 10, 20 and 30 nM, respectively), where the nuclei, actin filaments and tubulins were stained as blue, red and green, respectively.

(B, C) Cell spreading area and aspect ratio as a function of the PTX concentration, respectively. Data are shown from at least 3 independent experiments ($n \geq 3$) with mean ± SD. ns - no significant difference.

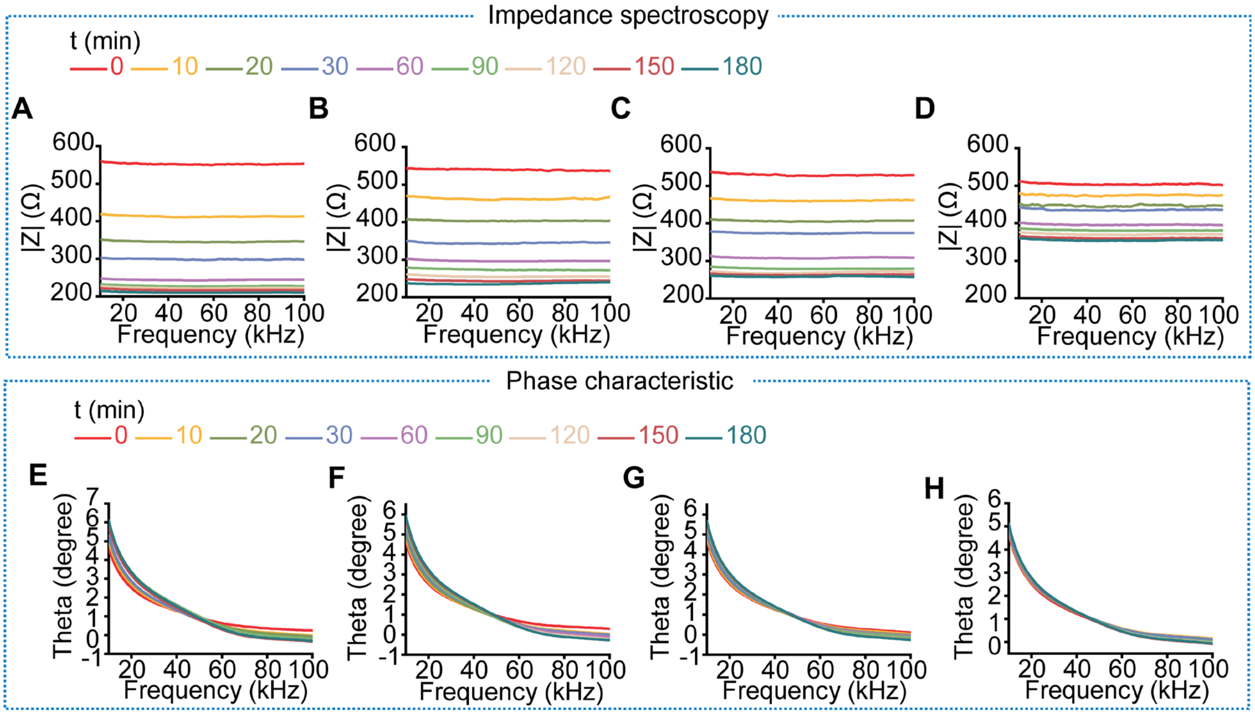

Figure S17. Representative results of 3D-CIMA for monitoring upward migration of collective cells pretreated with different concentrations of PTX ((A, E) 0 nM, (B, F) 10 nM, (C, G) 20 nM and (D, H) 30 nM) in the 2.5 mg/ml 3D Coll I matrix. 3D-CIMA data are presented as impedance (Ω) and phase (degree).

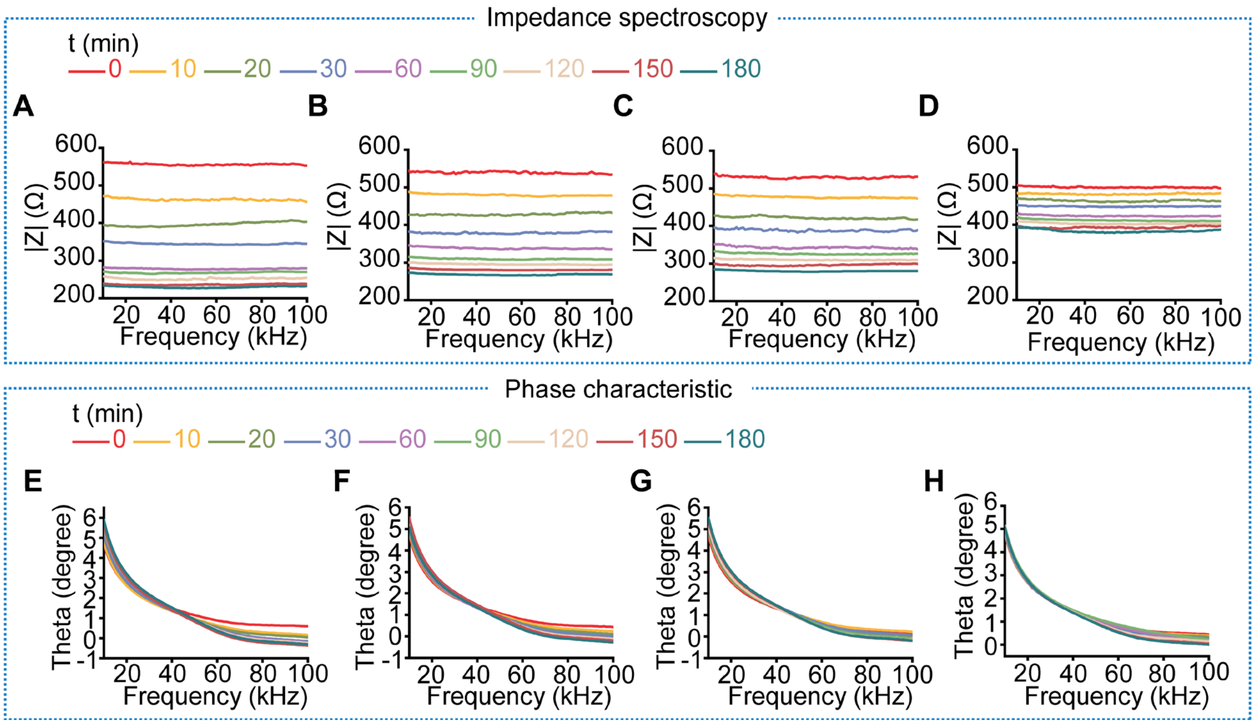

Figure S18. Representative results of 3D-CIMA for monitoring upward migration of collective cells pretreated with different concentrations of PTX ((A, E) 0 nM, (B, F) 10 nM, (C, G) 20 nM and (D, H) 30 nM) in the 3.0 mg/ml 3D Coll I matrix. 3D-CIMA data are presented as impedance (Ω) and phase (degree).

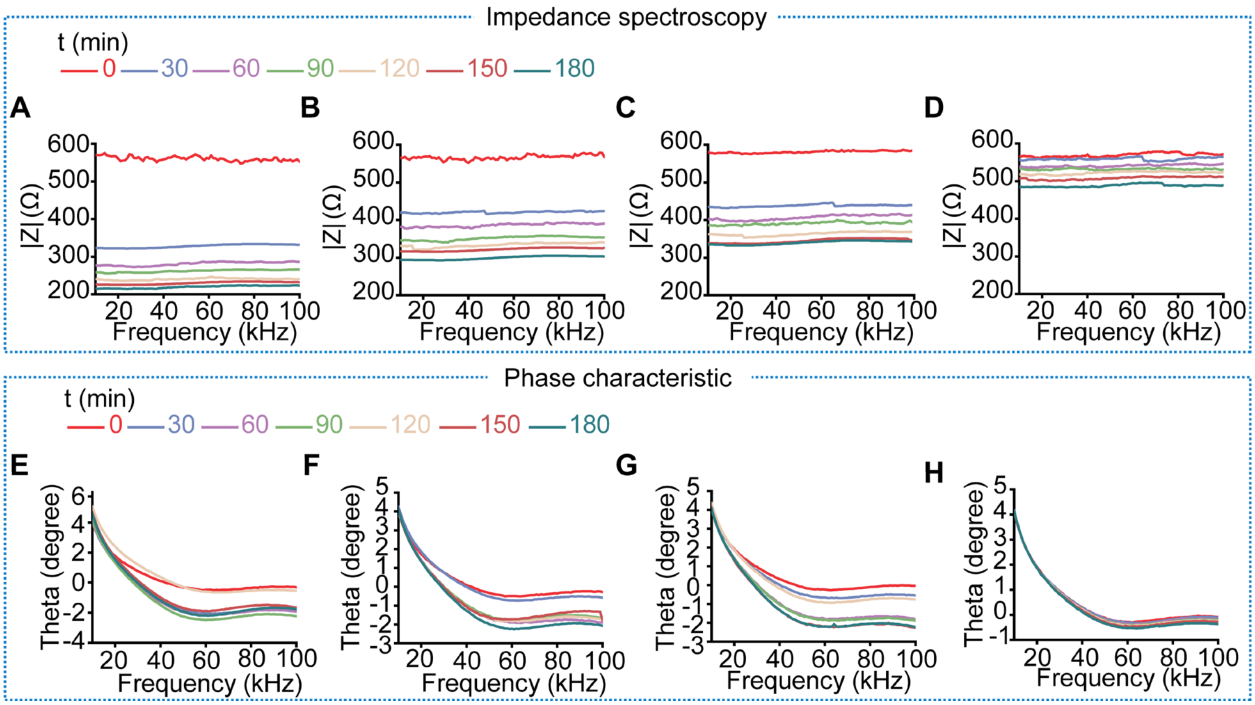

Figure S19. Representative results of 3D-CIMA for monitoring upward migration of collective cells pretreated with 20 μM GM6001 in 3D Coll I matrices of different monomer concentrations ((A, E) 2.5 mg/ml, (B, F) 3.0 mg/ml, (C, G) 3.5 mg/ml and (D, H) 4.0 mg/ml). 3D-CIMA data are presented as impedance (Ω) and phase (degree).

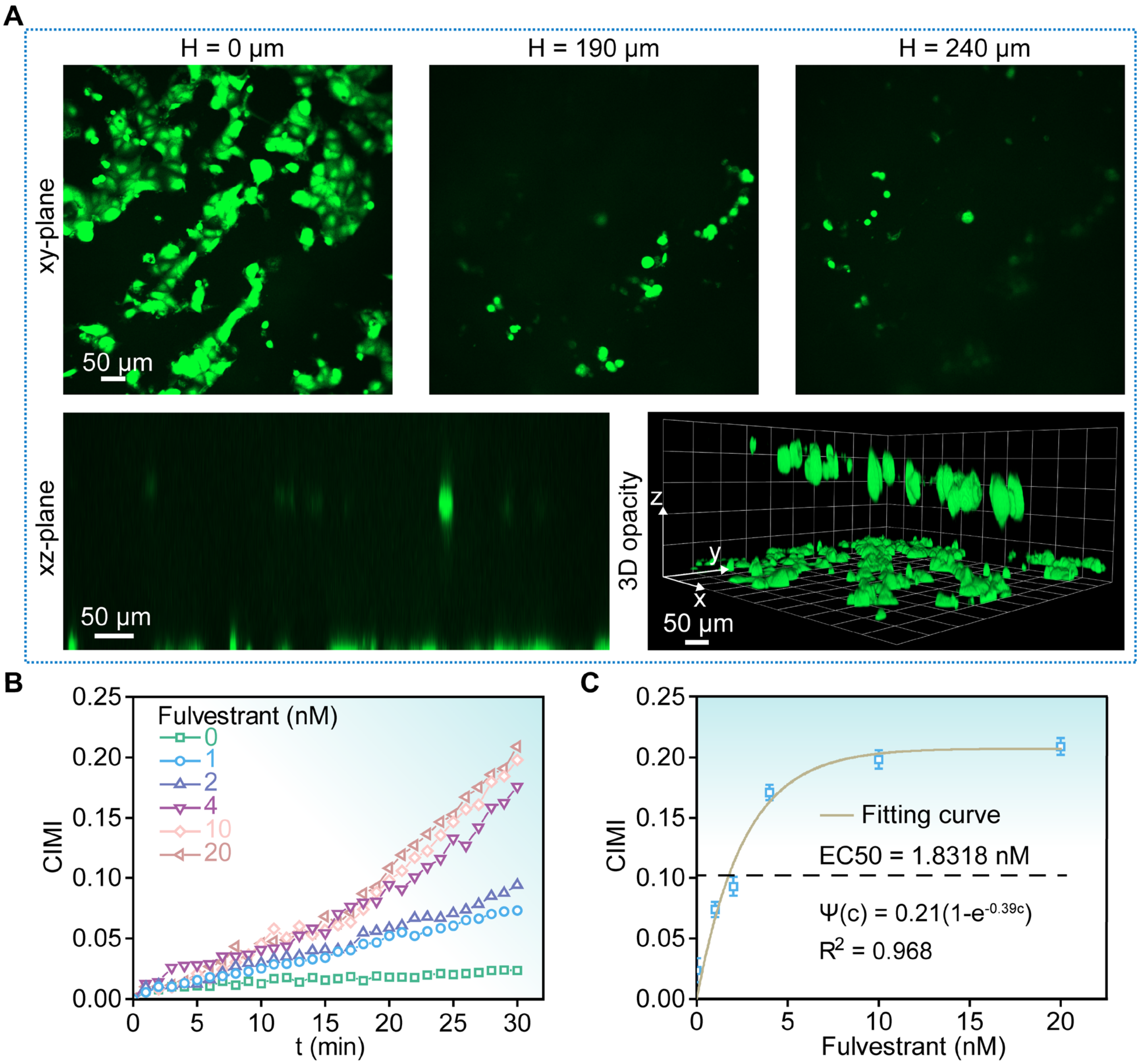

Figure S20. Loss of ERα induces the amoeboid migration of MCF-7 cells in the 3D collagen matrix.

(A) Spatiotemporal dynamics of MCF-7 cells invasion under the treatment of 10 nM fulvestrant, which was monitored by laser scanning confocal microscopy. The total invasion time was set as 180 min in the current experiments.

(B) Representative normalized signals (CIMI) of MCF-7 cells cultured in 3 mg/ml Coll Ⅰ matrix, which were recorded by 3D-CIMA in a real-time fashion. In the experiment, the cells were treated with fulvestrant of different concentrations (i.e., 0, 1, 2, 4, 10 and 20 nM).

(C) CIMI as a function of the fulvestrant concentration with EC50 = 1.8318 nM. Data are shown as Mean ± SD, n = 3.

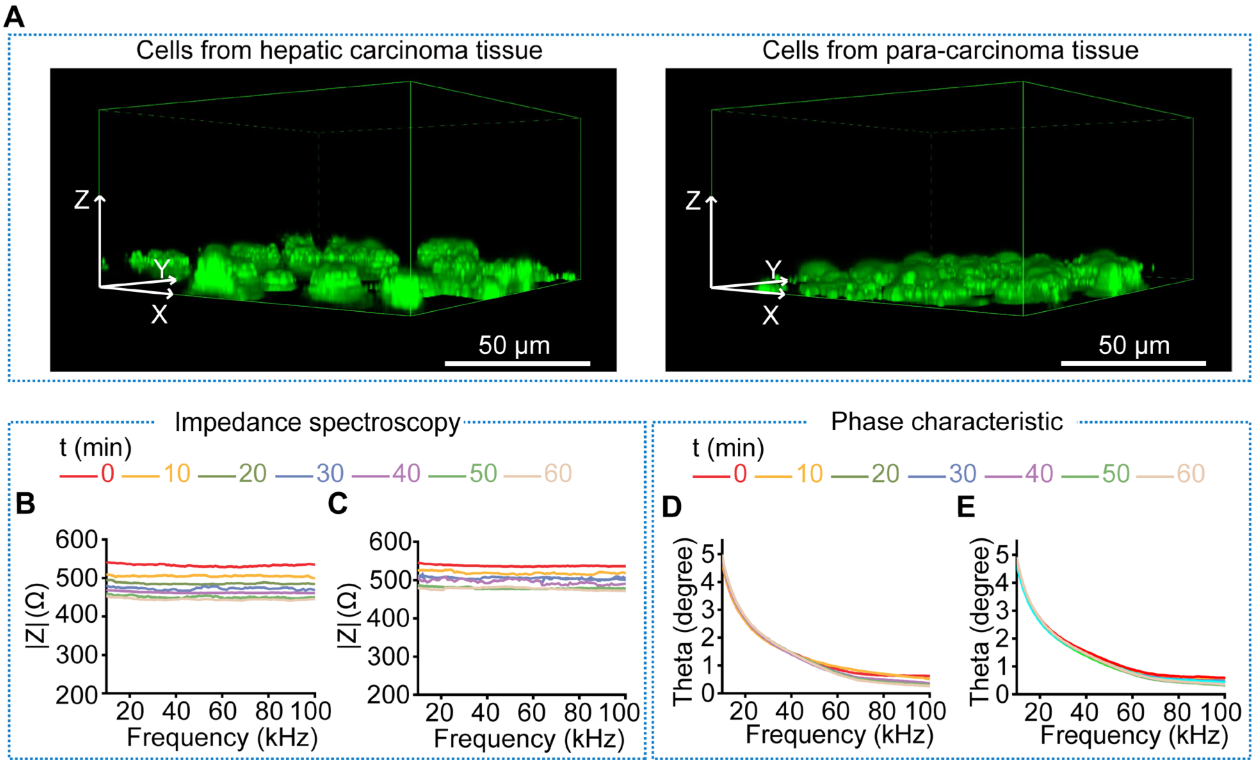

Figure S21. 3D invasion/migration assay based on cells from human hepatic carcinoma specimens.

(A) Spatiotemporal dynamics of cells from human hepatic carcinoma tissues and para-carcinoma tissues, monitored by laser scanning confocal microscopy. The total invasion time is set as 60 min in the current experiments.

(B-E) Representative results of 3D-CIMA, showing upward invasion/migration of collective cells from the hepatic carcinoma tissue (B, D) and para-carcinoma tissue (C, E) in the Coll I matrix of 2.5 mg/ml. These data are presented as impedance (Ω) and phase (degree), respectively.

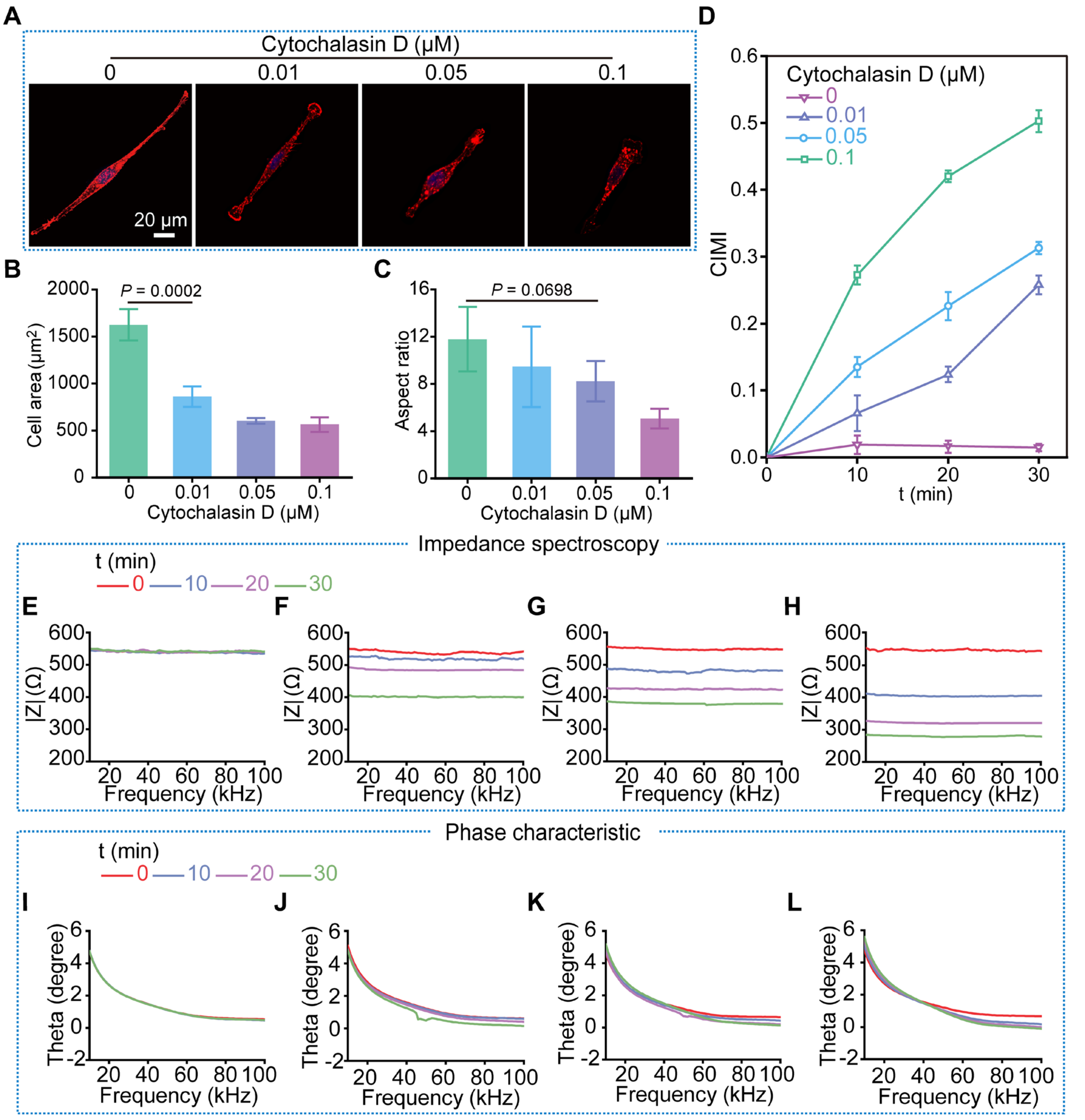

Figure S22. Changes in impedance spectroscopy for 2D adherent cells regulated by cytochalasin D.

(A) Representative images of single MDA-MB-231 cells pretreated with cytochalasin D (0, 0.01, 0.05 and 0.1 μM, respectively) for 30 min, where the nuclei and actin filaments were stained as blue and red, respectively.

(B) and (C) Spreading areas and aspect ratios of the cells treated with different concentrations of cytochalasin D, respectively. Data are shown from at least 3 independent experiments ($n \geqslant 3$) with mean ± SD.

(D) Time-dependent CIMI data (Mean ± SD, n = 3) for the cells pretreated with cytochalasin D (0, 0.01, 0.05 and 0.1 μM, respectively).

(E)~(L) Representative impedance data measured from the cells adhered onto 2D interdigitated electrodes, which were pretreated with different concentrations of cytochalasin D ((E, I) 0 μM, (F, J) 0.01 μM, (G, K) 0.05 μM and (H, L) 0.1 μM). Data are presented as impedance (Ω) and phase (degree), respectively.

Supplementary Tables

Table S1. Fabrication of 3D Coll I matrices with different Young's moduli.

3D Coll I matrix (mg/ml)	8.91 mg/ml Coll I stock solution (µl)	0.02 mol/l acetic acid (µl)	DMEM culture medium (µl)	10× PBS (µl)	0.5 mol/l NaOH (µl)
2.5	283	436	150	100	42.5
3.0	343	376	150	100	42.5
3.5	394	325	150	100	42.5
4.0	449	270	150	100	42.5

Table S2. Cell invasion/migration-induced changes in resistance and capacitance of the electrical elements in the chip chambers with 2.5 mg/ml Coll I matrix.

t (min)	R_{ccm} (Ω)	R_{ECM} (Ω)	C_{ECM} (×10^{-6} F)	R_{seal} (Ω)	R_{cell} (Ω)	C_{cell} (×10^{-6} F)	R_{ct} (Ω)	Q (×10^{-7})	n
0	281	298	2.59	532	604	5.96	276	1.14	0.37
10	190	237	2.60	493	580	6.68	223	1.12	0.36
30	103	184	4.76	433	433	12.9	198	1.02	0.29
60	62	59	5.60	362	386	24.4	183	0.93	0.26
90	55	11.5	6.20	330	352	48.8	169	0.82	0.24
120	34	11.3	10.2	253	317	80.5	184	0.76	0.27
150	29	9.7	13.4	135	162	97.3	185	0.73	0.27

Table S3. Cell invasion/migration-induced changes in resistance and capacitance of the electrical elements in the chip chambers with 3.0 mg/ml Coll I matrix.

t (min)	R_{ccm} (Ω)	R_{ECM} (Ω)	C_{ECM} (×10^{-6} F)	R_{seal} (Ω)	R_{cell} (Ω)	C_{cell} (×10^{-6} F)	R_{ct} (Ω)	Q (×10^{-7})	n
0	344	371	3.60	698	700	8.46	215	0.86	0.18
10	212	273	3.88	629	644	10.3	254	1.14	0.38
30	143	189	2.87	459	463	12.2	204	0.91	0.28
60	86	148	3.59	425	434	16.2	195	0.88	0.29
90	71	72	4.59	380	407	27.6	199	0.91	0.32
120	50	7.6	0.75	64	71	5.84	196	0.92	0.36
150	49	1.4	0.16	17	43	10.9	186	0.80	0.23

Table S4. Cell invasion/migration-induced changes in resistance and capacitance of the electrical elements in the chip chambers with 3.5 mg/ml Coll I matrix.

t (min)	R_{ccm} (Ω)	R_{ECM} (Ω)	C_{ECM} (×10^{-6} F)	R_{seal} (Ω)	R_{cell} (Ω)	C_{cell} (×10^{-6} F)	R_{ct} (Ω)	Q (×10^{-7})	n
0	286	358	4.88	948	955	20.2	266	1.15	0.31
10	232	277	3.40	659	662	14.0	251	1.14	0.39
30	137	181	3.48	548	558	15.5	249	1.22	0.63
60	104	147	3.06	244	252	14.8	205	0.94	0.33
90	82	129	5.21	246	251	23.3	201	0.89	0.29
120	69	59	5.44	227	244	34.3	202	0.91	0.30
150	82	1.3	0.23	26	55	11.0	175	0.75	0.21

Table S5. Cell invasion/migration-induced changes in resistance and capacitance of the electrical elements in the chip chambers with 4.0 mg/ml Coll I matrix.

t (min)	R_{ccm} (Ω)	R_{ECM} (Ω)	C_{ECM} (×10^{-6} F)	R_{seal} (Ω)	R_{cell} (Ω)	C_{cell} (×10^{-6} F)	R_{ct} (Ω)	Q (×10^{-7})	n
0	319	298	2.25	433	432	8.81	242	1.10	0.38
10	296	297	2.59	401	401	10.3	249	1.14	0.39
30	295	289	3.07	355	354	12.0	214	0.87	0.18
60	256	283	3.79	291	289	14.5	202	0.78	0.15
90	175	233	5.45	215	221	25.0	268	1.19	0.37
120	160	229	6.19	197	204	30.0	262	1.16	0.35
150	96	61	5.89	150	165	44.3	317	1.65	0.49

Supplementary videos

Video S1. Laser etching of the high-throughput ITO IDEA.

Video S2. Time-lapse imaging of the 3D reconstruction of collective cell migration in 3.0 mg/ml Coll I matrix. Stacked images were acquired over the Coll I matrix layer of 300 μm thickness using an inverted laser scanning confocal microscope equipped with 20× /NA 0.75 objective (Nikon A1, Japan).

Video S3. Time-lapse imaging of the xy-plane of the collective cell migration in 3.0 mg/ml Coll I matrix. Stacked images were acquired over the Coll I matrix layer of 300 μm thickness using the same microscope equipped with 20× /NA 0.75 objective (Nikon A1, Japan).
